# Understanding Gender Biases and Differences in Web-Based Reviews of Sanctioned Physicians Through a Machine Learning Approach: Mixed Methods Study

**DOI:** 10.2196/34902

**Published:** 2022-09-08

**Authors:** Julia Barnett, Margrét Vilborg Bjarnadóttir, David Anderson, Chong Chen

**Affiliations:** 1 Technology and Social Behavior Northwestern University Evanston, IL United States; 2 Robert H. Smith School of Business University of Maryland College Park, MD United States; 3 Villanova School of Business Villanova University Villanova, PA United States

**Keywords:** gender, natural language processing, web-based reviews, physician ratings by customer, text mining

## Abstract

**Background:**

Previous studies have highlighted gender differences in web-based physician reviews; however, so far, no study has linked web-based ratings with quality of care.

**Objective:**

We compared a consumer-generated measure of physician quality (web-based ratings) with a clinical quality outcome (sanctions for malpractice or improper behavior) to understand how patients’ perceptions and evaluations of physicians differ based on the physician’s gender.

**Methods:**

We used data from a large web-based physician review website and the Federation of State Medical Boards. We implemented paragraph vector methods to identify words that are specific to and indicative of separate groups of physicians. Then, we enriched these findings by using the National Research Council Canada word-emotion association lexicon to assign emotional scores to reviews for different subpopulations according to gender, gender and sanction, and gender and rating.

**Results:**

We found statistically significant differences in the sentiment and emotion of reviews between male and female physicians. Numerical ratings are lower and sentiment in text reviews is more negative for women who will be sanctioned than for men who will be sanctioned; sanctioned male physicians are still associated with positive reviews.

**Conclusions:**

Given the growing impact of web-based reviews on demand for physician services, understanding the different dynamics of reviews for male and female physicians is important for consumers and platform architects who may revisit their platform design.

## Introduction

### Background

Web-based reviews of physicians play an important role in patients’ searches for providers. A 2017 National Institutes of Health survey found that 39% of adults used web-based reviews to help them decide which physician to see [1]. Although ratings are widely used, they are also sparse, with many physicians having only 1 or 2 ratings on any given site. In addition, up to 90% of all web-based reviews are positive [2]. Positive reviews have been estimated to increase physician demand by as much as 7% [3]. Thus, to fully understand the impact of web-based reviews on individual providers and the health care system as a whole, it is important to identify when and why physicians are given negative reviews and whether those negative reviews actually reflect reality.

There are many reasons to believe that web-based reviews may be unhelpful. For example, they are subject to review fraud, as Hu et al [4] estimated that 10% of all web-based reviews are fraudulent. In addition, they are typically nonexpert reviews of expert services; that is, credence services, and customer reviews of credence services are generally thought to be unhelpful [5]. For both reasons, Dr Peter Carmel, president of the American Medical Association, has argued that “anonymous online opinions of physicians should be taken with grain of salt and should not be a patient’s sole source of information when looking for a new physician” [6]. Studies have further found that although web-based reviews matched peer evaluations of physicians in outpatient specialties, this was not the case for inpatient surgical specialties [7].

Gender adds another layer of complexity to questions related to the influence, validity, and value of web-based reviews. Studies have shown that female physicians are given more negative reviews and that women are rated as less amicable [8]. Although previous studies have shown differences in review content based on physician gender, our study adds a critical dimension by considering an external indicator of physician quality. We used data from the Federation of State Medical Boards on physicians who have been sanctioned by their state medical boards for unsuitability to practice medicine, either for negligence, malpractice, or other improper behavior. Sanctions range from probation to complete revocation of the offending physician’s medical license. As receiving a sanction is an objective marker of low-quality medical care, at least for some physicians, looking at sanctions gives us a way to quantify physician quality, which is a notoriously difficult task. We showed that women receive systematically different reviews from men and that female physicians who will be sanctioned in the future are rated lower and receive more negative comments in their reviews than similarly situated male physicians.

### Related Literature

#### Physician’s Gender

Previous studies have explored gender differences in light of how physicians consult and communicate with their patients. Studies have concluded that female physicians are generally more communicative and interpersonal than male physicians as they focus more on building partnership, asking questions, and providing information, which results in long medical appointments with female physicians [9]. This long consultation duration reduces the volume of consultations that female physicians can provide [10]. Some studies have explored the reasons for long consultations and the role of gender in medical decision-making. For instance, when diagnosing coronary heart disease, female physicians are more engaged with the historical presentation of the patient’s condition and more likely to be affected by the patient’s gender than male physicians. Greenwood et al [11] showed that female patients who had heart attacks treated by male physicians had significantly higher mortality than female patients treated by female physicians.

Web-based physician reviews have been studied from diverse angles. We divided our summary of the literature into review content, how reviews correlate with peer ratings, fraudulent reviews, sentiment analysis, and finally, the impact of physician reviews.

#### Content of Physician Reviews

Hao and Zhang [12] implemented latent Dirichlet allocation (LDA) as a topic modeling technique for textual review of data about Chinese physicians in 4 major specialty areas and identified popular review topics, including professionalism and showing appreciation for physicians’ detailed symptom descriptions [12]. A second study identified patient satisfaction, staff, and access as important themes in the reviews studied [13].

An extensive analysis of reviews from US health care review websites by Thawani et al [14] found that female physicians receive lower ratings overall, even after accounting for specialty. Comments about female physicians are more likely to be related to their interpersonal skills, whereas for male physicians, comments focus more on professionalism and helpfulness. Marrero et al [15] further examined a subset of the same data to understand the influence of gender on how patients both perceive and evaluate their surgeons, confirming that women are evaluated more positively for social interactions and men for technical aptitude.

#### Validity of Physician Reviews

McGrath et al [7] examined the validity of patient-generated web-based physician reviews and found that validity is affected by physician specialty. For specialties such as family medicine, allergies, internal medicine, and pediatrics, the web-based ratings of physicians listed as a *top doctor* by their peers are significantly higher than the ratings of those without this peer-generated quality indicator. Kordzadeh [16] showed that the ratings listed on hospital websites are systematically higher than those on outside commercial physician rating sites such as RateMDs and Google Reviews.

#### Sentiment of Physician Reviews

Wallace et al [17] developed a factorial LDA model to jointly identify both sentiment and topics from reviews. By incorporating the factorial LDA output into regression analysis, they further found that positive sentiment is associated with health care measurements such as patients’ revisit probability and health care costs and that the model can explain more variance than models using only rating information. Similarly, Rivas et al [18] developed a dependency tree–based classifier to capture patterns from each review, which can be used to sort physician reviews into a 2D classification system based on topic and polarity. Waltena et al [19] focused on the impact of sentiment on topic extraction in hospital reviews, and by adding 2 topics representing positive and negative sentiment in latent semantic analysis, the authors successfully reduced the bias owing to sentiment on the subjects of topics.

#### Impact of Physician Reviews

The impact of web-based physician reviews on patient choice remains an active area of research. Xu et al [3] explored the interaction between web-based physician reviews and physician demand and concluded that the number of reviews and disclosure of reviewer identity are positively related to physician demand but negatively correlated with review length. Through a counterfactual experiment, they found that strategies for improving ratings (eg, disclosing reviewers’ identities and limiting review length) can increase the demand for a physician by as much as 7.24%. However, improving the operational process or platform design can increase physician demand even further. Li et al [20] studied how web-based reviews and physicians’ gender affect patients’ primary care physician choices. The results indicated that among physicians whose skills are endorsed in reviews, if a female physician is endorsed for their interpersonal characteristics, such as compassion and personableness, they are more likely to be chosen than a male physician endorsed for the same reasons. However, this kind of gender effect is not observed among physicians endorsed for their technical skills. Bedside manner, diagnosis accuracy, patients’ waiting time, and consultation length are critical in patients’ choice of a physician [3].

Our study is the first to analyze the content of patient reviews of physicians across genders using natural language processing tools that accounts for differences in ratings and sanction status. This allowed us to understand both the set of criteria on which male and female physicians are evaluated and the impact of poor performance (as measured by sanctions). We further applied an emotional index to understand, in a multidimensional way, the tones of the different types of reviews based on ratings, gender, and sanction status. More specifically, in this study, we aimed to determine whether reviews of women systematically differ from those of men. In particular, we aimed to discover whether female physicians are rated lower at baseline than male physicians and whether female physicians experience larger reputational penalties than male physicians for low-quality services (as indicated by sanctions from the state medical board).

## Methods

### Data

Our data were collected from 2 sources: physician reviews were obtained from RateMDs and combined with physician sanction data from the Federation of State Medical Boards [21].

The data from RateMDs include physicians’ average ratings on a 1- to 5-star scale. Reviewers rate the overall experience and 4 other defined categories: helpfulness, knowledgeability, punctuality, and staff. The data further contain the text of the reviews.

State licensing boards issue sanctions to physicians for issues related to their suitability to practice medicine in each state. Reasons for sanctions include, but are not limited to, serious malpractice, performing unnecessary treatment, fraudulent billing, and abuse of patients. We collected every review posted between October 2004 and August 2011 and matched it by name, location, state, and specialty with the database of sanctioned physicians from the Federation of State Medical Boards. We removed any reviews of physicians that were made after they were sanctioned, so any official sanction does not affect the content of the reviews. In total, we obtained 403,470 reviews of 134,973 physicians across the United States. In our data, men were more than twice as likely as women to be sanctioned; 1.7% (1629/95,831) of all male physicians were sanctioned, whereas only 0.64% (250/39,142) of female physicians were sanctioned.

The web-based reviews from RateMDs were merged with the state medical board sanction data by matching physician name (including matching using a dictionary of common nicknames [eg, Kate for Katherine]), state, specialty, medical school, and graduation year (where available). The physicians in the sanction data who we could not perfectly match owing to multiple matches or no matches (and which amounted to <5% of the sample) were excluded from the study.

### Methodology

#### Overview

The field of text mining and natural language processing is growing rapidly, with many emerging techniques available to analyze text and discover patterns in documents via automated procedures. In their book, *Foundations of Statistical Natural Language Processing*, Manning and Schutze [22] stated that the availability of large text corpora has changed the scientific approach to language in linguistics and cognitive science. Therefore, phenomena that were previously undetectable or seemingly uninteresting have become the central focus of lexical analysis. Taking advantage of some of these new developments, in this study, we implemented paragraph vector (as described in the following sections) and used a word-emotion association lexicon on the corpus of physician reviews to analyze the data in a nuanced manner.

#### Data Preprocessing

To make the raw data analyzable, we performed a series of tasks. First, the reviews were converted to lowercase, so that capital letters are treated the same as lowercase letters. Second, punctuation was removed because it typically adds unnecessary noise to word models. Third, *stopwords*, defined as unimportant words that are overly common (eg, “the,” “and,” and “is”) were removed using a freely available System for the Mechanical Analysis and Retrieval of Text stopword list built by Salton and Buckley and sourced from web-based Appendix 11 of the paper by Lewis et al [23]. Fourth, we removed numbers because, similar to punctuation, they add noise to the analysis. On the remaining words in the corpus, we performed *stemming* using the Porter stemming algorithm [24,25]. Stemming is the act of reducing words to their root form (eg, “practice,” “practicing,” and “practiced” become “practic”). This allows models to treat these words as one concept rather than as separate ideas. As we had a limited-sized data set, we applied all the preprocessing steps mentioned previously to maximize insights from a concise vocabulary. Although the removal of stopwords resulted in some locally unnatural word sequences (such as articles not appearing before nouns), we found that this did not hinder our analysis.

#### Analytical Techniques

In this study, we applied a paragraph vector framework [26], a natural language processing method that represents each word or document as a dense vector (ie, a location in 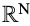 space), called an embedding, which is then used as an input to train a model to predict co-occurrence of words. We used the paragraph vector distributed bag-of-words model, which uses words from a given width window to predict the next word in the document. In this framework, “kind” is located closer to “nice” than “surgery” because “nice” has a much higher probability than “surgery” of being found in similar contexts as “kind.” We used the paragraph vector model to generate an embedding of words, which can be used to calculate the similarity (via cosine similarity) between any set of words or documents. Henceforth, we refer to the cosine similarity between words or documents as the *similarity score*.

For each data slice (eg, sanctioned physicians), we trained a paragraph vector model. Once the model was trained, we could use the embedding to identify words associated with the medical reviews of different types of physicians (eg, based on gender). To compare specific differences across a physician population, we concatenated every review from one specific subset of data (eg, sanctioned *male* physicians) and found the similarity scores of this document with each word within the corpus. Then, we repeated this process for the complementary subset (eg, sanctioned *female* physicians) and compared the similarity scores for each subset. We extracted the words with the largest differences between the subsets. For example, we computed the similarity of *wait* to the female corpus of reviews, computed the similarity of *wait* to the male corpus of reviews, and calculated the difference in these scores. Our analysis focused on the words with the highest absolute difference between the similarity scores for one subset of reviews (typically female) and the complementary subset (typically male).

To understand the *emotional* nature of the reviews, we used the NRC word-emotion association lexicon [27] to attribute sentiment and emotional scores to the corpus (NRC stands for the National Research Council Canada, but the lexicon is commonly referred to as the NRC emotion lexicon). This lexicon created an *afinn* dictionary by rating words on a scale of 8 emotions: anger, anticipation, disgust, fear, joy, sadness, surprise, and trust. Using the scores from this lexicon, we were able to both rate reviews on an aggregate emotional scale (how *emotional* the document is as a whole) and rank them for each of the 8 emotions. More specifically, for each data cut (eg, sanctioned female physicians), each word in each physician’s review was scored based on the emotional score of the word, and then, average physician score was derived by averaging all physicians’ emotional scores. Understanding these emotional scores allowed us to develop a deep understanding of the criteria that patients use to evaluate female and male physicians and how those criteria differ.

**Figure 1:**
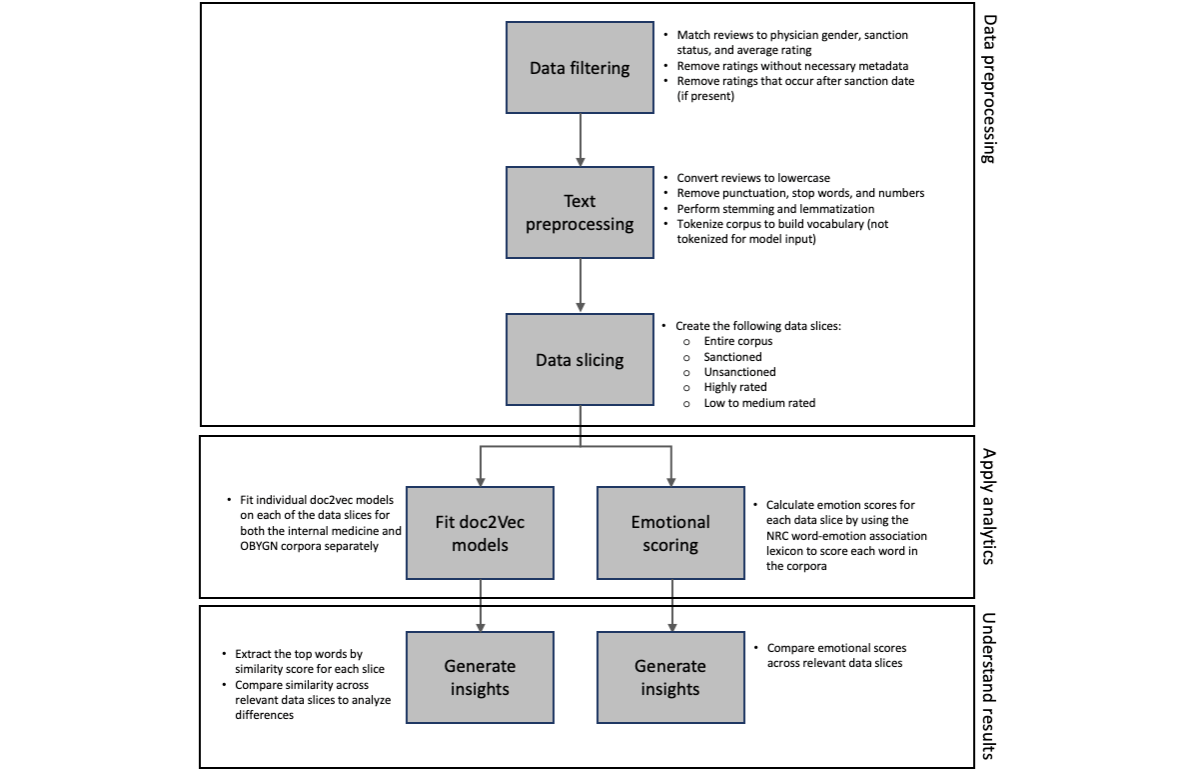
Analysis flowchart. NRC: National Research Council Canada; OBGYN: obstetrics and gynecology.

We have summarized the methodological approach in Figure 1.

#### Implementation and Hypertuning

We used doc2vec [28], a Python package implementing paragraph vectors, to learn how patients review physicians differently across gender, sanctions, and ratings (both in isolation and interaction). We performed a standard doc2vec implementation to learn the paragraph vectors of the following:

1. Gender (male and female)
2. Composite label of gender and sanction (female sanctioned, female unsanctioned, male sanctioned, and male unsanctioned)
3. Composite label of gender and rating (female high rating, female low to medium rating, male high rating, and male low to medium rating), where we defined high rating as ≥4 stars and low to medium rating as <4 stars

To overcome majority bias, we sampled an equal number of reviews for each group. We trained the models independently for the different metadata cuts, rather than treating each separate review as an individual document. By fitting the different groups separately, we were able to understand the specific lexicons associated with each metadata cut (gender, sanction, and rating). Then, we analyzed the similarity scores of words to their respective corpora and compared the scores.

We pretrained the paragraph vector framework, using the continuous bag-of-words algorithm to tune the hyperparameters, by testing the most similar words to several words such as “knowledgeable,” “wonderful,” “caring,” and “rude.” We ran multiple variations of the model to identify the best settings. The results were consistent across different parameters, which gave us confidence in the robustness of the final model. We have listed the exact parameter settings in Multimedia Appendix 1 and summarized the results of the paragraph vector model for “knowledgeable,” “wonderful,” “caring,” and “rude” in Figure S2 in Multimedia Appendix 1.

### Ethical Considerations

All the data used in this study are publicly available and do not contain identifiable private information about individuals. Thus, this study was not deemed to require institutional review board review. After merging sanction data with review data by name, specific physician identities were removed from the data set and not used in the analyses.

## Results

### Data Overview

**Figure 2:**
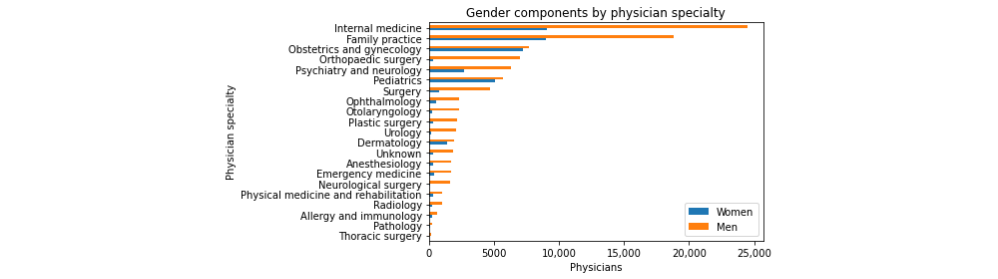
Number of physicians in each specialty, broken down by gender.

Figure 2 shows the number of physicians in each specialty by gender. Internal medicine and family practice are the 2 most common specialties in our data. The figure highlights that there are more male physicians than female physicians in every specialty; overall, 29% (39,142/134,973) of the physicians in the sample are women. This gender imbalance is easily noticeable in the more common disciplines; internal medicine has the highest number of female physicians, but there are still twice as many male physicians. The imbalance is even more prominent in some small disciplines such as orthopedic surgery and neurological surgery, where men outnumber women 23:1 and 15:1, respectively. Obstetrics and gynecology (OBGYN) and pediatrics departments are more balanced in terms of gender, with an approximately even ratio of men to women.

Table 1 highlights the average star ratings overall and for each of the main categories present in the reviews (helpfulness, knowledgeability, punctuality, and staff). This pattern is consistent across specialties including internal medicine and OBGYN. Furthermore, unsanctioned physicians receive higher ratings than sanctioned physicians. We note that the *staff* category ratings may not be reflective of the physician’s medical capabilities. In all cases, the average rating for men is higher than that for women. These differences are statistically significant (*P*<.001, evaluated with 2-tailed *t* tests) both when comparing unsanctioned male physicians with unsanctioned female physicians and when comparing unsanctioned female or male physicians with sanctioned female or male physicians. When the ratings of sanctioned male physicians are compared with those of sanctioned female physicians, the absolute differences are of similar magnitude; however, owing to the small size of the sanctioned population, the differences are not statistically significant. Among sanctioned physicians, female physicians receive lower ratings (by an average of approximately 0.1 stars) than male physicians (not considering specialties). The difference between genders among sanctioned physicians is greater than that among unsanctioned physicians, especially for those rated around average for helpfulness and knowledgeability. A detailed breakdown of the number of sanctioned physicians is provided in Table S1 in Multimedia Appendix 1.

**Table 1:** Average star rating (out of 5 stars) overall and for the 4 RateMDs score categories for the whole sample of physicians. Ratings are separated by gender and sanction status.

Categories	Full sample (n=134,973), mean (SD)	Female physicians (n=39,142, 29%), mean (SD)			Male physicians (n=95,831, 71%), mean (SD)	
		Unsanctioned (n=38,892, 99.36%)	Sanctioned (n=250, 0.64%)	Unsanctioned (n=94,202, 98.30%)		Sanctioned (n=1629, 1.69%)
Overall	3.86 (1.12)	3.81 (1.12)	3.41 (1.21)	3.89 (1.12)		3.52 (1.24)
Helpfulness	3.89 (1.36)	3.85 (1.35)	3.47 (1.44)	3.90 (1.36)		3.59 (1.48)
Knowledgeability	4.03 (1.25)	3.99 (1.24)	3.62 (1.35)	4.06 (1.25)		3.75 (1.38)
Punctuality	3.83 (1.18)	3.77 (1.18)	3.39 (1.27)	3.86 (1.18)		3.43 (1.31)
Staff	3.67 (1.3)	3.61 (1.3)	3.02 (1.41)	3.70 (1.3)		3.19 (1.45)

On average, each physician receives 3 reviews, with an average length of 55.7 (SD 47.65) words. In general, lower-ranked physicians receive longer reviews than higher-ranked physicians; people have more to say about an experience they are dissatisfied with. On average, women receive longer reviews than men. An exception is in the OBGYN field—in this specialty, patients have more to say about a sanctioned male physician than they do about a sanctioned female physician. However, on the whole and across specialties, the group with the longest reviews are the low to medium–ranked women. Given what we know from our subsequent content analysis, this is because patients have longer and more negative comments to make about women, whereas reviews of male physicians are short and more positive. As highlighted in Table 2, these trends hold when we break down the length analysis by specialty.

**Table 2:** Average review length for sanctioned and unsanctioned male physicians and female physicians in all specialties, internal medicine, and OBGYN^a^, measured in number of words.

Categories	All specialties (n=134,973)		Internal medicine (n=33,549)		OBGYN (n=15,001)	
	Female (n=39,142, 29%), mean (SD)	Male (n=95,831, 71%), mean (SD)	Female (n=9087, 27.09%), mean (SD)	Male (n=24,462, 72.91%), mean (SD)	Female (n=7268, 48.45%), mean (SD)	Male (n=7733, 51.55%), mean (SD)
Overall	50.1 (36.4)	45.7 (36.1)	45.8 (36.3)	41.5 (35.2)	58 (34.6)	54.5 (34.9)
Sanctioned	48.7 (36.6)	47.6 (37.1)	55.1 (34)	42.9 (35.8)	46 (30.8)	51.2 (38.7)
Unsanctioned	50.2 (36.4)	45.6 (36.1)	45.7 (36.3)	41.4 (35.2)	58.2 (34.7)	54.5 (34.9)
High rating	39 (31)	37.2 (31.3)	35.9 (31)	33.4 (29.8)	45.4 (29.6)	47.8 (32)
Low to medium rating	61.6 (38)	56.3 (38.8)	57.3 (38.5)	53 (38.9)	69.7 (34.8)	65 (36.7)

^a^OBGYN: obstetrics and gynecology.

The nature of physicians’ work differs between specialties, which in turn may influence web-based reviews. Therefore, to remove the impact of specialty, our analysis in this study focused on internal medicine (the most common type of physician reviewed). In addition, we conducted the analysis on OBGYN reviews and compared the results with those for internal medicine (detailed results for the OBGYN reviews are available in Multimedia Appendix 1). This allowed us to compare results across medical specialties, but the OBGYN results are particularly interesting, as we can be confident that most reviews are written by women, giving us further insight into the differences in results. Following these analyses, we compared the length and emotion of the reviews.

We examined the differences between gender and reviews in 3 ways: first, we analyzed male and female physicians; second, we studied both gender and rating; and third, we analyzed the interaction of sanction and gender. For each of these analyses, a separate doc2vec model was trained on the relevant corpus (eg, the entire corpus, reviews of sanctioned physicians, or highly ranked reviews). For each analysis (eg, male physician vs female physician in highly ranked reviews), we extracted the top words by similarity score to the paragraph vector of concatenated female reviews and concatenated male reviews, respectively, in the relevant subset of data, and then compared the differences. Our analysis focused on the words with the greatest absolute difference between the similarity scores for female and male reviews. Additional and complementary results are available in Multimedia Appendix 1.

### Review Comparison Between Genders

**Figure 3:**
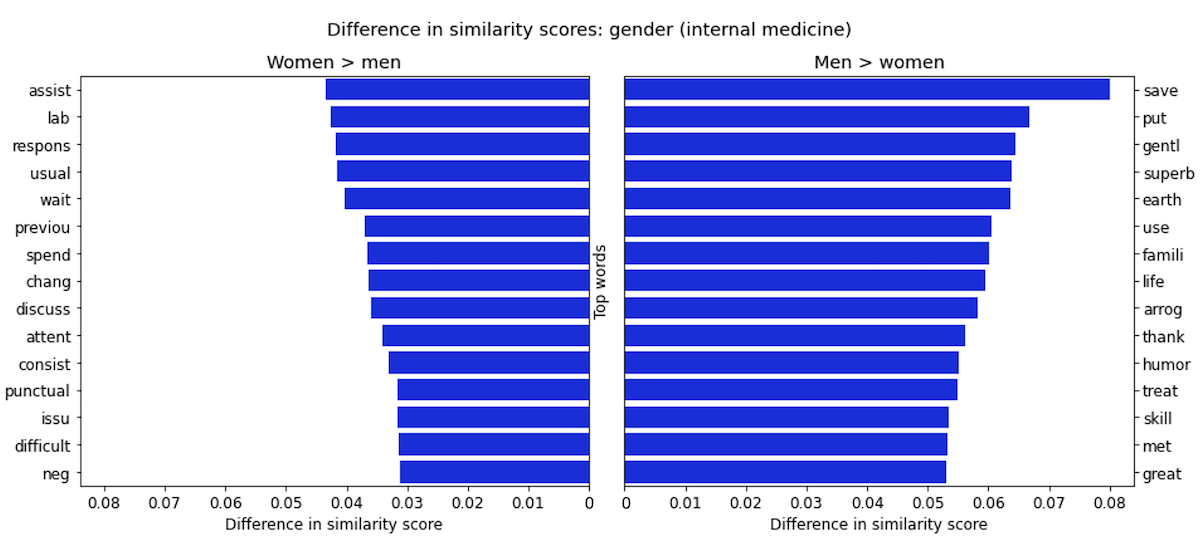
Difference in similarity scores for top words in reviews of male and female internal medicine physicians. The x-axis represents the absolute difference in similarity score for the given words to the document vector of concatenated reviews for all women and all men. The figure displays the top 15 words; the biggest differences in similarity scores are for the female subset of reviews over male reviews (left pane) and the male subset of reviews over female reviews (right pane).

To examine the relative similarity scores of the words used in the corpus to describe men and women, we extracted the top words by similarity score (omitting procedural-type words, eg, “appt” and “said” for analysis purposes) for the subset of male physician reviews and female physician reviews, as summarized in Figure 3. This figure presents the top 15 words with the largest difference between similarity scores to the document vector for concatenated female reviews and concatenated male reviews, with the left pane showing the 15 words that scored highest for the female reviews and the right pane showing those that scored highest for male reviews.

This led us to several interesting observations. For example, “assist,” “neg,” “difficult,” “wait,” “punctual,” and “issue” were scored as more similar to female physicians’ reviews than male physicians’ reviews. In contrast, “superb,” “gentl,” “famil,” “skill,” “humor,” and “great” were scored as more similar to male physicians’ reviews. The key takeaway is that even before we incorporate sanction and ranking data, we see stark differences between the ways male and female physicians are evaluated, which supports the findings of previous analyses [14-16]. From this general comparison, without considering rating, we see that women are more often evaluated with a focus on punctuality, whereas men are much more likely to be praised for their technical abilities and bedside manner. To confirm that these underlying frequencies are statistically significant, we performed a chi-square test for the top words presented in Figure 3 (the null hypothesis was that there is no difference in these frequencies, and the alternative hypothesis was that there are differences in these frequencies not equal to 0) and found all of them to be significant (*P***<**.001).

### Review Comparison Between Gender and Rating

**Figure 4:**
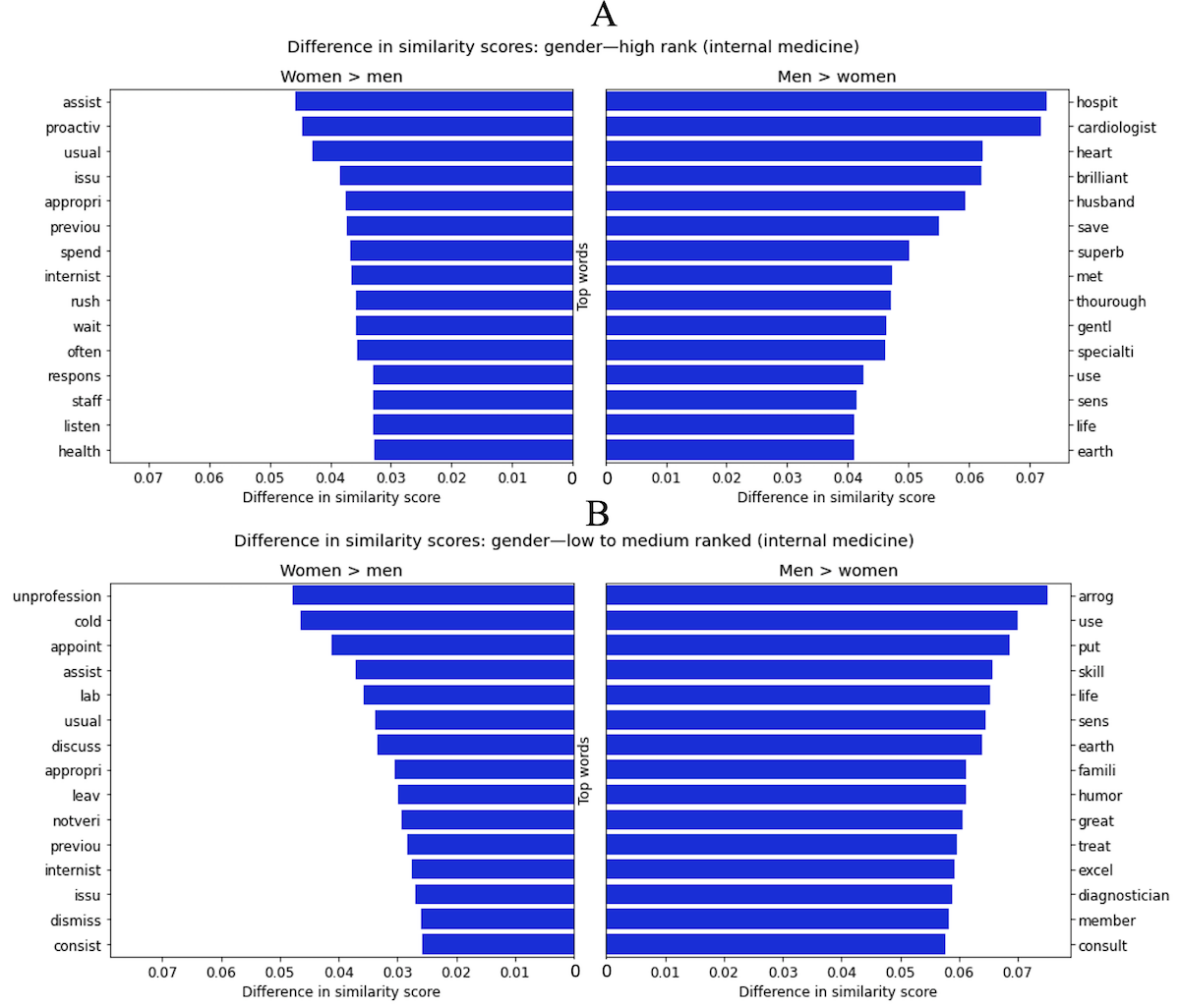
Difference in similarity scores for top words for (A) high-ranked and (B) low to medium–ranked men and women in internal medicine. The x-axis represents the absolute difference in similarity score for the given words to the document vector of concatenated reviews for all (A) high-ranked women and all men and (B) low to medium–ranked women and all men. The figure displays the top 15 words with the biggest differences in similarity scores for the female subset of reviews over male reviews (left pane) and the male subset of reviews over female reviews (right pane).

We used the approximate mean as the standard criterion for determining whether a rating was *high* (>4 stars) or *low to medium* (≤4 stars). Then, we created document vectors for the following subsets of concatenated reviews: (1) reviews rating female physicians highly, (2) reviews rating male physicians highly, (3) reviews rating female physicians as medium to low, and (4) reviews rating male physicians as medium to low. We repeated this analysis, focusing first on high-ranked female and male physicians and second on low to medium–ranked female and male physicians. The results are shown in Figure 4. We again compared the top words by absolute difference in similarity score between men and women within the high reviews first and then within the low to medium reviews.

For highly ranked physicians, the words that are the most associated with female physicians’ reviews over the corpora or male physicians’ reviews tend to either describe the timeliness of the visit (eg, “wait” and “rush”), liken female physicians to workers in supporting roles, or evaluate staff in those supporting roles (eg, “assist” and “staff”). In contrast, the corpora of male physicians’ reviews are more likely to contain words that are medically technical (eg, “hospit,” “cardiologist,” “skill,” or “diagnostician”) or simply glowing endorsements (eg, “brilliant,” “superb,” and “greatest”). These findings are summarized in Figure 4A.

Despite these discrepancies, we note that highly ranked physicians generally garner positive text reviews regardless of gender. Gender differences become much more pronounced when focusing on low-ranked physicians. As summarized in Figure 4B, the words with the highest similarity scores for reviews for low to medium–ranked women are objectively much more negative (eg, “unprofession,” “cold,” “issu,” “dismiss,” and “notveri”) compared with the reviews of low to medium–ranked men (eg, “skill,” “sens,” “famili,” “humor,” “great,” and “excel”). The only objectively negative word that is much more likely to occur in these male physicians’ reviews is “arrog” for arrogance (a quality more often attributed to men than to women).

### Review Comparison Between Gender and Sanction

**Figure 5:**
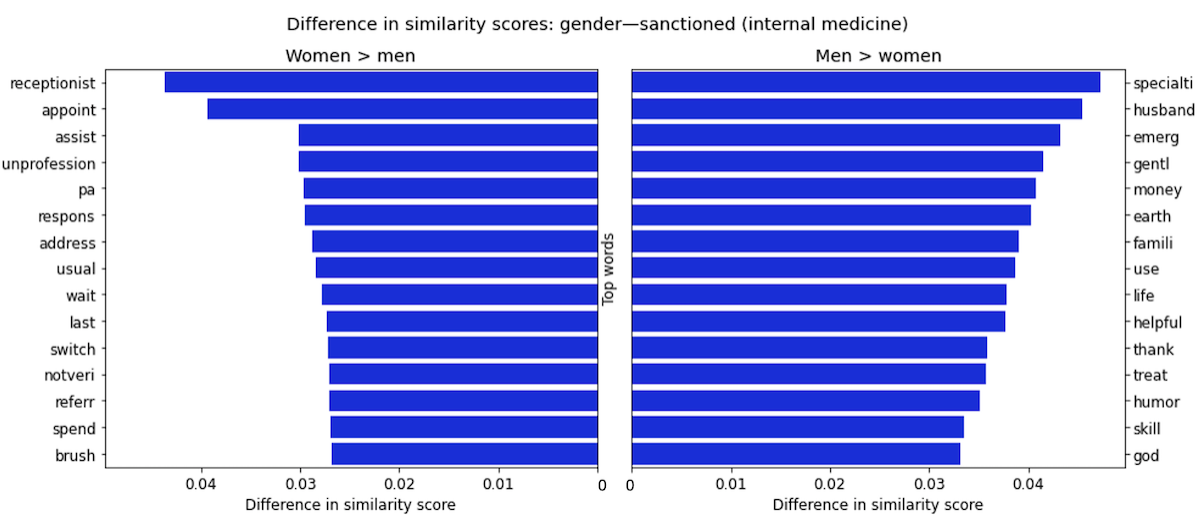
Difference in similarity scores for top words for sanctioned men and women in internal medicine. The x-axis represents the absolute difference in similarity score for the given words to the document vector of concatenated reviews for all sanctioned women and all sanctioned men. The figure displays the top 15 words with the biggest differences in similarity scores for the female subset of reviews over male reviews (left pane) and the male subset of reviews over female reviews (right pane).

As discussed previously, male physicians receive high ratings on average, but at the same time are more likely to be sanctioned. This motivated our independent analysis of reviews of sanctioned and unsanctioned physicians by gender. Owing to the low overall probability of sanctions (1879/134,973, 1.39% of our sample), the reviews of unsanctioned physicians mirror the general discrepancies between men and women. In contrast, the analysis of sanctioned physicians’ reviews reveals stark gender differences, as highlighted in Figure 5. The words with the highest probability of appearing in sanctioned women’s reviews have much more negative connotations than those in sanctioned men’s reviews, whereas it is much more difficult to tell the difference between a sanctioned man and an unsanctioned man. Some of the words most highly associated with sanctioned male physicians are “specialti,” “gentl,” “helpful,” “thank,” “skill,” and “god,” whereas some of the highest scored words for sanctioned female physicians are “receptionist,” “unprofession,” “pa,” “wait,” and “notveri.” Words that are exclusive to the sanctioned male lexicon include “cardiologist,” “save,” “heart,” “hospit,” “superb,” “pleasur,” and “compassion,” which highlight the stark discrepancies even further because these words do not appear even once in a sanctioned female physician’s review (additional details are available in Figures S4 and S5 in Multimedia Appendix 1).

### Emotion Scoring

We analyzed the emotional scores of the reviews, generating an emotional score for each subset of physicians. We used the percentage of each top word appearing in each cut of the review corpus multiplied by the emotional score, repeated the process for each word in the lexicon to obtain a total score for each cut (eg, male, female, sanctioned women, and highly rated men), and then summed these scores within each subset. The emotion analysis is the only portion of this study in which we found noticeable differences between the 2 specialties analyzed—internal medicine and OBGYN. Therefore, we have included the results for both specialties in the main text.

In the plots below, the emotions are categorized as positive, negative, or neutral and listed alphabetically within each category in the following order: joy, positive, trust, anticipation, surprise, anger, disgust, fear, negative, and sadness.

**Figure 6:**
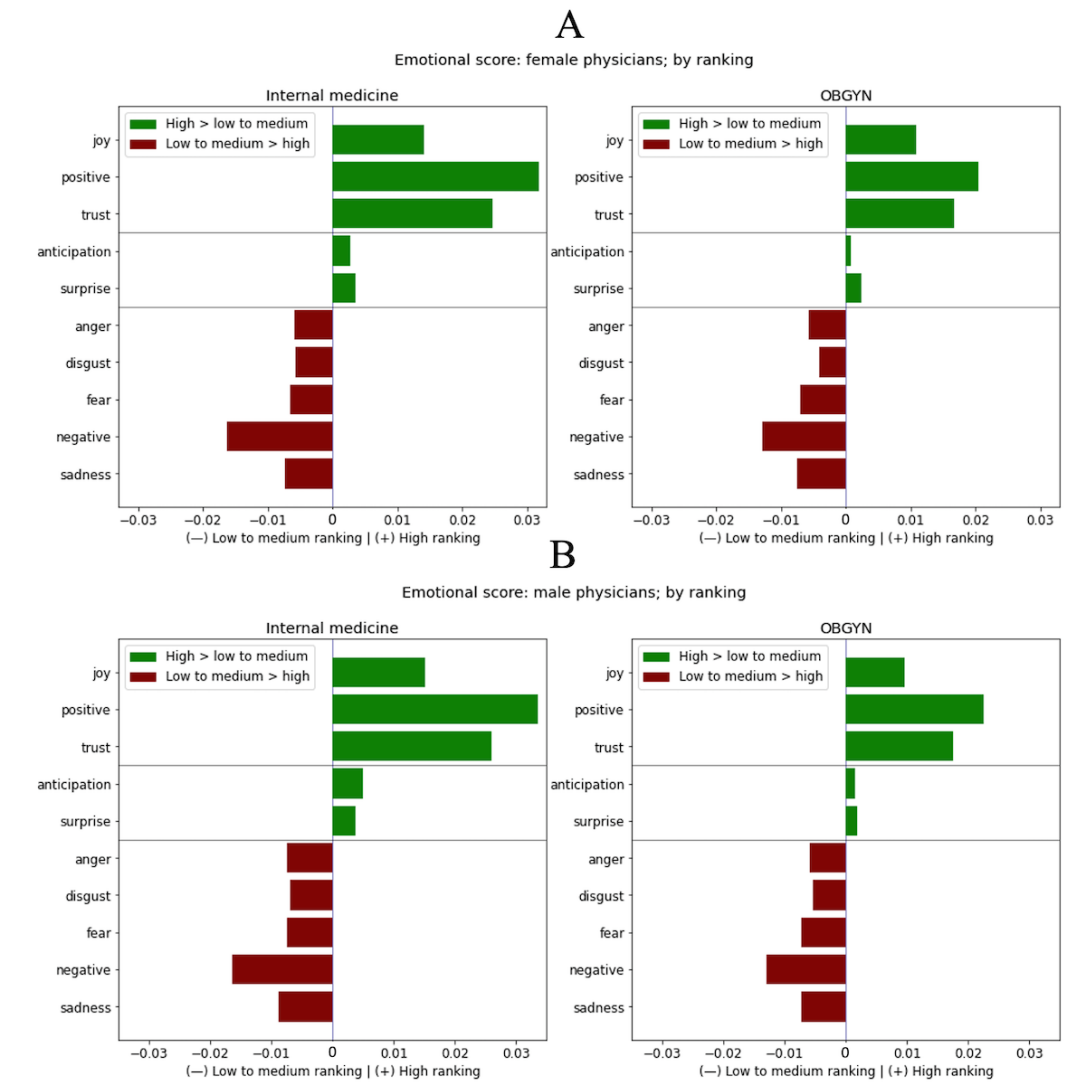
Emotional score ratings for (A) female physicians’ and (B) male physicians’ reviews. The 10 emotions on the y-axis are categorized as positive, neutral, or negative (and arranged alphabetically within these categories). The x-axis plots the difference in the emotional score between the different groups. Positive numbers mean that an emotion scored high for high-ranked physicians, and negative numbers mean the emotion scored high for low to medium–ranked physicians. OBGYN: obstetrics and gynecology.

First, we examined the differences in emotional scores between high-ranked and low to medium–ranked female physicians (Figure 6A) and between high-ranked and low to medium–ranked male physicians (Figure 6B). As expected, more positive emotions are much more likely to be found in high ratings of both men and women, with only small differences between men and women in both specialties analyzed.

**Figure 7:**
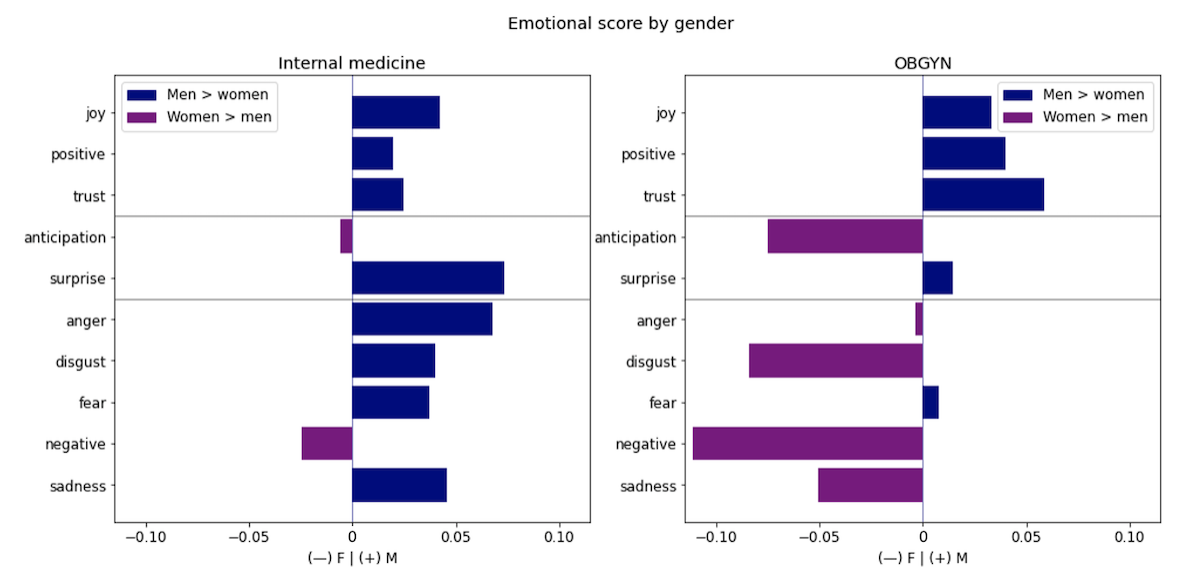
Emotional scores by gender for internal medicine and obstetrics and gynecology (OBGYN).

When using gender (rather than rating) as the main dimension of analysis, we found that for internal medicine, reviews of men are much more emotional than those of women, for both positive and negative emotions, as demonstrated in Figure 7. A notable exception is that women’s reviews scored high on negative emotion. For OBGYN physicians (reviews that we can safely assume to be written mostly by women), the reviews are much more positive for men (overindexing on joy, positive, and trust), and the reviews of female physicians score notably high on anticipation, disgust, negativity, and sadness.

**Figure 8:**
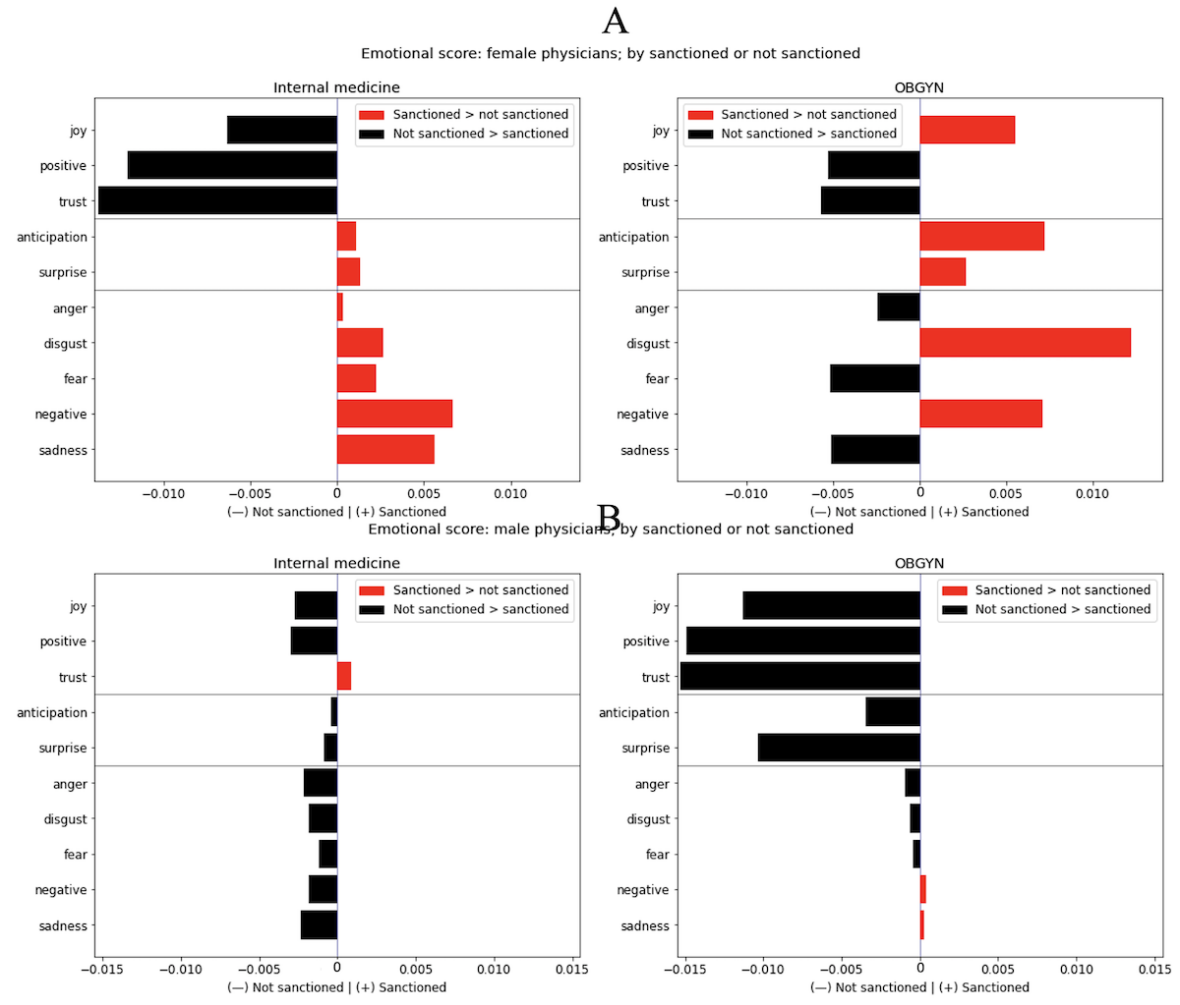
Emotional scores by sanction status for (A) female physicians and (B) male physicians for both internal medicine and obstetrics and gynecology (OBGYN).

Next, we divided the analysis by gender and specialty, and then focused on the difference between sanctioned and unsanctioned physicians. The results are highlighted in Figures 8A and 8B. For female internal medicine physicians, the results are consistent with expectations; unsanctioned physicians score high on positive emotions, whereas sanctioned physicians score high on neutral and negative emotions. In contrast, for male internists, unsanctioned physicians score high across the emotional scale (however, the differences are generally small). The pattern for OBGYN physicians is very different—among female OBGYN physicians, there is great variability in the emotional scores, whereas among male OBGYN physicians, unsanctioned physicians score high on positive and neutral emotions, with very little difference in emotional scores on negative emotions.

Finally, we focused on the differences between sanctioned men and sanctioned women and summarize the results in Figure S18 in Multimedia Appendix 1. In contrast to Figure 8, where we hold the gender constant and analyze across sanction statuses, in Figure S18 in Multimedia Appendix 1, we hold the sanction status constant and analyze across genders. This shows the differences in reviews for both sanctioned women and men and unsanctioned women and men. For both specialties, we found small gender differences among unsanctioned physicians. However, among sanctioned physicians, the differences are large: women, especially female internists, are disproportionately reviewed in a more negative manner. Patients in the OBGYN department tend to review sanctioned male physicians more emotionally, in both positive and negative terms (with the exception of disgust and negativity, for which sanctioned female OBGYN physicians score high on average).

Overall, we conclude that emotional scoring analysis adds a layer of depth to our understanding of the differences among lexical reviews of physicians. The differences between the specialties are even more fascinating—although we are unable to discern major differences between the specialties regarding general word composition of the lexicons, the emotional discrepancies between internal medicine reviews (written by a mix of patients) and OBGYN reviews (written by mostly female patients) are extremely clear. Holding everything else constant, internal medicine reviews of male physicians tend to be largely more emotional, regardless of whether that emotion is positive or negative. Reviews of female OBGYN physicians tend to be much more negative.

When we add sanction status to the analysis, the dynamic becomes more complex. In internal medicine, there are no notable differences in the emotional scores of sanctioned men and unsanctioned men. In contrast, reviews of sanctioned female physicians in internal medicine show negative emotion more prominently than those of unsanctioned female physicians. The biggest difference between emotional scores in this entire analysis is between unsanctioned and sanctioned male OBGYN physicians—sanctioned male OBGYN physicians receive the most negative reviews in any subset of data analyzed. When comparing sanctioned women directly with sanctioned men, sanctioned female internal medicine physicians are reviewed much more negatively than sanctioned male internal medicine physicians, but reviews of sanctioned male OBGYN physicians are more emotional overall, regardless of whether the emotion is positive or negative.

## Discussion

### Overview

In this study, we analyzed web-based reviews of physicians and how they differ based on physicians’ gender. We further sought to understand the complex interaction among the physician’s web-based score (rating), whether they are sanctioned by a state medical board, and gender, as revealed in the content of the web-based reviews. To investigate this interaction, we implemented paragraph vector techniques to identify words that are specific to and indicative of the separate metadata cuts. Then, we enriched these findings by using the NRC word-emotion association lexicon to assign emotional scores to 3 segments: gender, gender and sanction, and gender and rating.

### Principal Findings

Our findings shed light on the different criteria by which patients evaluate male and female physicians, and they highlight the disparity in severity with which patients review male and female physicians. When we analyze the ratings of male and female physicians while holding the rating range constant, it becomes clear that women are more likely to be evaluated on their interpersonal bedside manner, whereas men are more likely to be evaluated based on their perceived technical skills and performance. This pattern holds when analyzing reviews of low-rated or medium-rated male physicians—the lexical content of their reviews is still much more likely to convey high praise, whereas women are much more likely to be severely criticized. The dynamic is further exacerbated among men and women who are sanctioned. It is much more difficult to discern a review of a sanctioned man from the review of an unsanctioned man by the content of the written review alone, whereas for women, there is a stark contrast, and female physicians are evaluated much more harshly if they are sanctioned. The insight gained by analyzing sanctioned physicians is an important contribution of this study. There are baseline differences between how male and female physicians are perceived, but those differences are greatly magnified when the service quality is low. Sanctioned men still receive glowing reviews, whereas sanctioned women experience large reputational penalties when they deliver low-quality care or behave inappropriately.

It is essential to understand not only the quantitative differences in how and why female and male physicians are evaluated but also the qualitative aspect of those differences. Contributing to this qualitative understanding, our findings elucidate the gender-driven difference in bases for evaluations of physicians by patients. Most notably, we did not see differences in the emotional language used for sanctioned and unsanctioned male physicians, whereas female physicians who will be sanctioned have consistently more negative emotion associated with their reviews.

### Comparison With Previous Studies

An expanding stream of literature shows significant gender bias in ratings, perhaps most egregiously in a case in which changing the name of an anonymous teaching assistant from male to female lowered the average review score [29]. Our study contributes to the growing literature on how web-based medical reviews are biased by gender, highlighting that in web-based reviews, women are more likely to receive negative reviews, obtain low scores, and be judged on criteria not directly related to their skills as a physician (eg, diagnostic abilities) [20,21]. We make a unique contribution by examining how physicians who are sanctioned for inappropriate behavior, negligence, or malpractice are penalized for low-quality service.

### Limitations

Our results are subject to a few limitations imposed by the data. First, we only have review data and do not know the actual quality of care delivered (except care by sanctioned physicians, which we know is more likely to be poor). We do not know the types of services received, and we do not know the patient outcomes. We tried to account for these unknowns by averaging all patient reviews for each physician and comparing physicians within subspecialties, which should control for much of the variation in services provided. However, if the medical services provided within a subspecialty systematically differ between genders, there may still be some residual confounding. Second, our data set does not contain physicians’ race or ethnicity, which is another potential dimension of review bias. Future studies can investigate the possibility of racial and gender bias. Third, in our data, sanctioned physicians’ reviews before the sanction date were combined; therefore, we could not explore the commonality or information signals provided by no-text reviews or by the length of individual reviews. Fourth, owing to the small number of sanctioned physicians, we represented the presence or absence of sanctions with a binary indicator; however, sanction severity varies. Therefore, future studies can focus on sanction severity to provide a more detailed and nuanced analysis of reviews. Finally, we acknowledge that the data are a decade old at the time of publication, meaning that if there have been sociological changes in patients’ views and behavior related to physician’s gender, our results will not capture those recent developments.

### Conclusions

The role and influence of web-based reviews may grow as medicine becomes increasingly computerized, a shift that has only been accelerated by the COVID-19 pandemic. As telemedicine expands in scope and prevalence, proximity becomes less of a limiting factor in selecting a physician; therefore, patients will rely more on web-based reviews to guide their physician choices. Given this growing role of reviews in physician selection, action needs to be taken to ensure that they are fair and balanced. Although awareness is the first step, websites and apps that feature or contain physician reviews should also follow best practices for mitigating gender and racial bias in those reviews. For instance, as previous studies have shown, asking specific questions rather than providing open-ended boxes for reviews can reduce bias [30]. Similarly, highlighting the potential for unconscious bias [31] and providing a rubric for evaluations [32] can also help web-based platforms to mitigate biases in physician reviews.

## Appendix

Multimedia Appendix 1 Additional results, charts, and tables.

## Abbreviations

LDA: latent Dirichlet allocation
NRC: National Research Council Canada
OBGYN: obstetrics and gynecology

## References

[1] Holliday AM, Kachalia A, Meyer GS, Sequist TD (2017). Physician and patient views on public physician rating websites: a cross-sectional study. J Gen Intern Med.

[2] Emmert M, Sander U, Pisch F (2013). Eight questions about physician-rating websites: a systematic review. J Med Internet Res.

[3] Xu Y, Armony M, Ghose A (2021). The interplay between online reviews and physician demand: an empirical investigation. Manag Sci.

[4] Hu N, Liu L, Sambamurthy V (2011). Fraud detection in online consumer reviews. Decis Support Syst.

[5] Lantzy S, Anderson D (2020). Can consumers use online reviews to avoid unsuitable doctors? Evidence from rateMDs.com and the Federation of State Medical Boards. Decis Sci.

[6] Lieber R (2012). The Web Is Awash in Reviews, but Not for Doctors. Here’s Why. The New York Times.

[7] McGrath RJ, Priestley JL, Zhou Y, Culligan PJ (2018). The validity of online patient ratings of physicians: analysis of physician peer reviews and patient ratings. Interact J Med Res.

[8] Dunivin Z, Zadunayski L, Baskota U, Siek K, Mankoff J (2020). Gender, soft skills, and patient experience in online physician reviews: a large-scale text analysis. J Med Internet Res.

[9] Roter D, Lipkin Jr M, Korsgaard A (1991). Sex differences in patients' and physicians' communication during primary care medical visits. Med Care.

[10] Jefferson L, Bloor K, Birks Y, Hewitt C, Bland M (2013). Effect of physicians' gender on communication and consultation length: a systematic review and meta-analysis. J Health Serv Res Policy.

[11] Greenwood BN, Carnahan S, Huang L (2018). Patient-physician gender concordance and increased mortality among female heart attack patients. Proc Natl Acad Sci U S A.

[12] Hao H, Zhang K (2016). The voice of Chinese health consumers: a text mining approach to Web-based physician reviews. J Med Internet Res.

[13] López A, Detz A, Ratanawongsa N, Sarkar U (2012). What patients say about their doctors online: a qualitative content analysis. J Gen Intern Med.

[14] Thawani A, Paul MJ, Sarkar U, Wallace BC (2019). Are online reviews of physicians biased against female providers?. Proceedings of the 4th Machine Learning for Healthcare Conference.

[15] Marrero K, King E, Fingeret AL (2020). Impact of surgeon gender on online physician reviews. J Surg Res.

[16] Kordzadeh N (2019). Investigating bias in the online physician reviews published on healthcare organizations' websites. Decis Support Syst.

[17] Wallace BC, Paul MJ, Sarkar U, Trikalinos TA, Dredze M (2014). A large-scale quantitative analysis of latent factors and sentiment in online doctor reviews. J Am Med Inform Assoc.

[18] Rivas R, Montazeri N, Le NX, Hristidis V (2018). Automatic classification of online doctor reviews: evaluation of text classifier algorithms. J Med Internet Res.

[19] Wartena C, Sander U, Patzelt C (2019). Sentiment independent topic detection in rated hospital reviews. Proceedings of the 13th International Conference on Computational Semantics - Short Papers.

[20] Li S, Lee-Won RJ, McKnight J (2019). Effects of online physician reviews and physician gender on perceptions of physician skills and Primary Care Physician (PCP) selection. Health Commun.

[21] (2018). Physician Data Center Query. Federation of State Medical Boards.

[22] Manning CD, Schutze H (1999). Foundations of Statistical Natural Language Processing.

[23] Lewis DD, Yang Y, Rose TG, Li F (2004). Rcv1: a new benchmark collection for text categorization research. J Mach Learn Res.

[24] Rijsbergen CJ, Robertson SE, Porter MF (1980). New Models in Probabilistic Information Retrieval. Vol. 5587.

[25] Porter MF (1980). An algorithm for suffix stripping. Program.

[26] Le Q, Mikolov T (2014). Distributed representations of sentences and documents. Proceedings of the 31st International Conference on Machine Learning.

[27] Mohammad SM, Turney PD (2013). Crowdsourcing a word-emotion association lexicon. Comput Intell.

[28] Lau JH, Baldwin T An empirical evaluation of doc2vec with practical insights into document embedding generation. arXiv..

[29] MacNell L, Driscoll A, Hunt AN (2014). What’s in a name: exposing gender bias in student ratings of teaching. Innov High Educ.

[30] Castilla EJ (2008). Gender, race, and meritocracy in organizational careers. AJS.

[31] Peterson DA, Biederman LA, Andersen D, Ditonto TM, Roe K (2019). Mitigating gender bias in student evaluations of teaching. PLoS One.

[32] Uhlmann EL, Cohen GL (2005). Constructed criteria: redefining merit to justify discrimination. Psychol Sci.

