# Supplementary material for: Understanding Gender Biases and Differences in Web-Based Reviews of Sanctioned Physicians Through a Machine Learning Approach: Mixed Methods Study
Source: JMIR Form Res. 2022 Sep 8;6(9):e34902. doi: 10.2196/34902 (PMC9501672; doi:10.2196/34902)
Supplement: Multimedia Appendix 1 [file formative_v6i9e34902_app1.pdf]

S Appendix

S1 Number of Sanctioned Doctors

Table S1 highlights that relatively few doctors are sanctioned, with most working in internal medicine, family practice, obstetrics and gynecology, and pediatrics, as these are the most common specialties. In all, approximately 0.7% of doctors in our sample were sanctioned.

	All Doctors		Male		Female	
**Specialty**	**Doctors**	**% Sanctioned**	**% Male**	**% Sanc.**	**% Female**	**% Sanc.**
Full Sample	134,973	1.02%	71%	1.17%	29%	0.64%
**Internal Medicine**	33,549	0.62%	73%	0.72%	27%	0.34%
**Family Practice**	27,853	1.41%	68%	1.69%	32%	0.82%
**OBGYN**	15,001	1.13%	52%	1.29%	48%	0.96%
**Pediatrics**	10,765	0.38%	53%	0.46%	47%	0.29%
**Psychiatry + Neurology**	9,009	1.17%	70%	1.38%	30%	0.67%
**Orthopaedic Surgery**	7,277	1.11%	96%	1.15%	4%	0.33%
**Surgery**	5,478	1.08%	86%	1.17%	14%	0.51%
**Dermatology**	3,325	1.05%	59%	1.13%	41%	0.94%
**Ophthalmology**	2,933	0.65%	80%	0.64%	20%	0.70%
**Otolaryngology**	2,582	1.08%	89%	1.22%	11%	0.00%
**Plastic Surgery**	2,504	1.48%	88%	1.60%	12%	0.64%
**Unknown**	2,243	2.36%	84%	2.55%	16%	1.40%
**Urology**	2,237	0.72%	93%	0.77%	7%	0.00%
**Emergency Medicine**	2,108	1.14%	81%	1.12%	19%	1.24%
**Anesthesiology**	2,051	2.15%	85%	2.24%	15%	1.62%
**Neruological Surgery**	1,738	1.90%	94%	1.96%	6%	0.93%
**Physical Medicine**	1,364	1.03%	77%	1.14%	23%	0.64%
**Radiology**	1,258	0.24%	80%	0.30%	20%	0.00%
**Allergy + Immunology**	913	0.55%	73%	0.75%	27%	0.00%
**Pathology**	381	0.52%	69%	0.38%	31%	0.83%
**Thoracic Surgery**	214	1.87%	93%	2.00%	7%	0.00%
**Preventive Medicine**	97	1.03%	73%	1.41%	27%	0.00%
**Colon + Rectal Surgery**	77	0.00%	86%	0.00%	14%	0.00%
**Medical Genetics**	9	0.00%	56%	0.00%	44%	0.00%
**Nuclear Medicine**	7	0.00%	100%	0.00%	0%	0.00%

Figure S1: Number of doctors by specialty in sample, and the breakdown of each specialty by gender and sanction status.

S2 Similarity Scores of “Base” Words in Pre-Trained Model

Hyperparameter tuning is an integral part of training doc2vec models. As there is no objective measure of general word similarity, we used human judgement to select the parameters that work the best on selected baseline words. These words were caring, rude, wonderful and knowledgeable. We find the best hyper-parameter setting producing interpretable results are vector size of 300, window size of 30, minimum counts of 30, and negative sampling drawing 7 noise words. Based on this setting, we set the hyper-parameter of doc2vec to be ($dm = 0, window = 30, vector\_size = 300, min\_count = 30, epochs = 100, workers = 4, hs = 0, negative = 7, dbow\_words = 1, dm\_concat = 1$). Figure S2 highlights the fitted similarity scores to base words for the selected parameter setting. The similarity scores indicate that the model operates well on words with standard definitions and synonyms.

Most similar words to 'caring'	
Words	Similarity score
'compassionate'	0.4045
'kind'	0.4009
'wonderful'	0.3906
'best'	0.3710
'excellent'	0.3534
'amazing'	0.3526
'cares'	0.3460
'dedicated'	0.3347
'always'	0.3220
'outstanding'	0.3064

Most similar words to 'rude'	
Words	Similarity score
'unprofessional'	0.4293
'unfriendly'	0.4199
'rudest'	0.4092
'awful'	0.3668
'horrible'	0.3625
'nasty'	0.3585
'office'	0.3223
'incompetent'	0.3088
'worst'	0.3026
'unhelpful'	0.3025

Most similar words to 'wonderful'	
Words	Similarity score
'great'	0.6361
'excellent'	0.5710
'amazing'	0.5622
'fantastic'	0.5557
'best'	0.4370
'outstanding'	0.4319
'kind'	0.4296
'love'	0.4259
'awesome'	0.3963
'caring'	0.3906

Most similar words to 'knowledgeable'	
Words	Similarity score
'knowledgable'	0.8640
'knowlegable'	0.4976
'knowlegeable'	0.4937
'intelligent'	0.4648
'competent'	0.4061
'knowledgeble'	0.3754
'smart'	0.3406
'informative'	0.3226
'knowledge'	0.3130
'knowledable'	0.2818

Figure S2: Predicted cosine similarity scores of base words.

S3 Additional Outputs from Internal Medicine

In the main text we evaluated the difference in cosine similarity scores to the document vectors associated with concatenated reviews for female and male physicians for the following different data slices: the entire corpus, reviews with high rankings, reviews with low-medium rankings, sanctioned doctors, and unsanctioned doctors. Below are the outputs with a different metric: words exclusive to the different cuts of data. In A2-6 we have the top words (ranked by cosine similarity score to the female corpus and male corpus) exclusive to female and male doctors in (A2) the entire corpus, (A3) highly ranked reviews, (A4) low to medium ranked reviews, (A5) sanctioned doctors, and (A6) unsanctioned doctors.

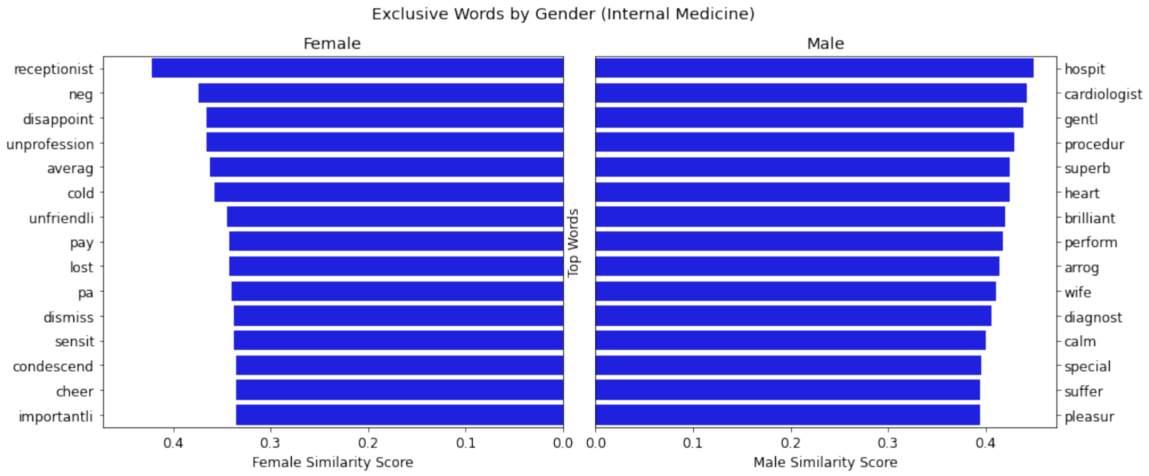

Figure S3: Top words exclusive to men and women in internal medicine. The x-axis represents the cosine similarity score between all of the words listed on the y categorical axis and the female subset of reviews (left pane) and male subset of reviews (right pane).

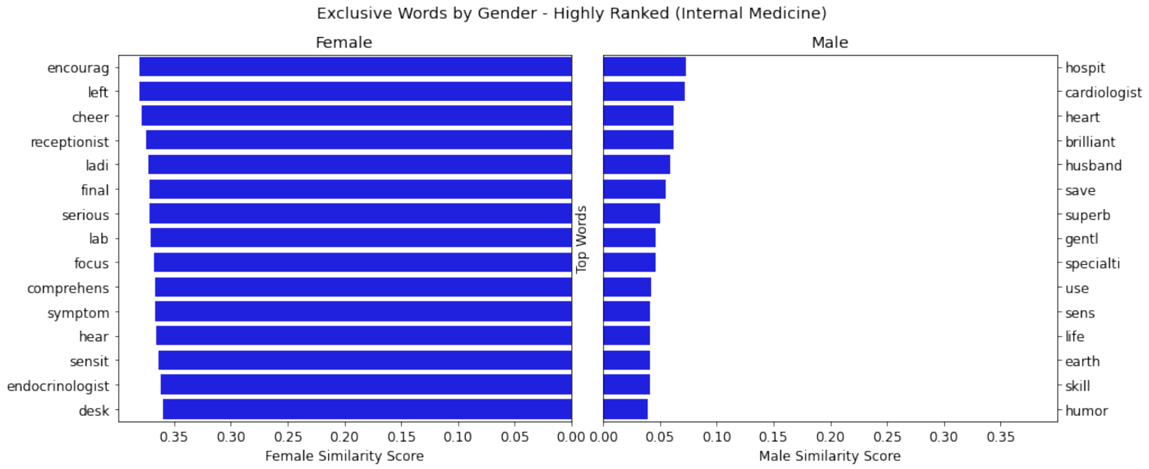

Figure S4: Top words exclusive to highly ranked men and women for men and women in internal medicine. The x-axis represents the cosine similarity score between all of the words listed on the y categorical axis and the female subset of reviews (left pane) and male subset of reviews (right pane).

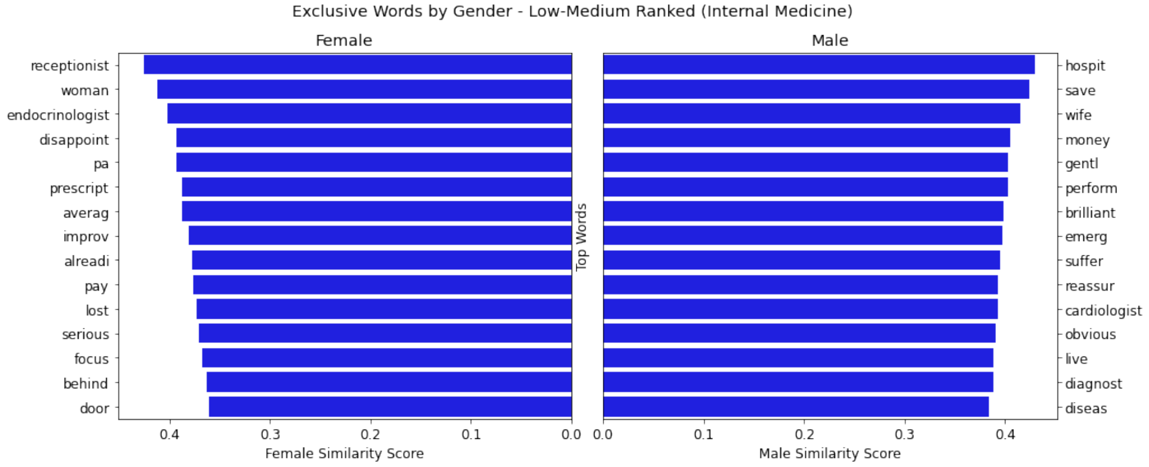

Figure S5: Top words exclusive to low-medium ranked men and women for men and women in internal medicine. The x-axis represents the cosine similarity score between all of the words listed on the y categorical axis and the female subset of reviews (left pane) and male subset of reviews (right pane).

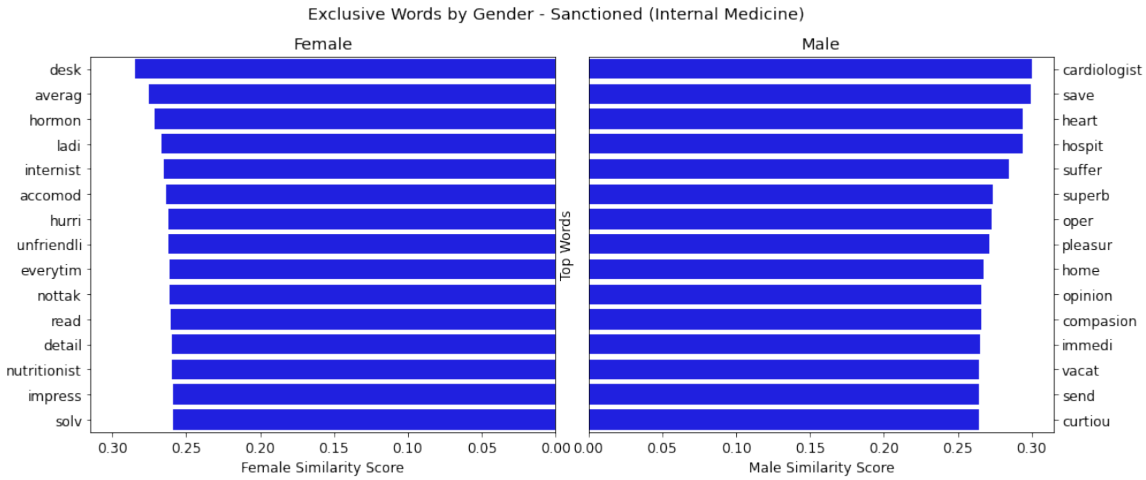

Figure S6: Top words exclusive to sanctioned men and women for men and women in internal medicine. The x-axis represents the cosine similarity score between all of the words listed on the y categorical axis and the female subset of reviews (left pane) and male subset of reviews (right pane).

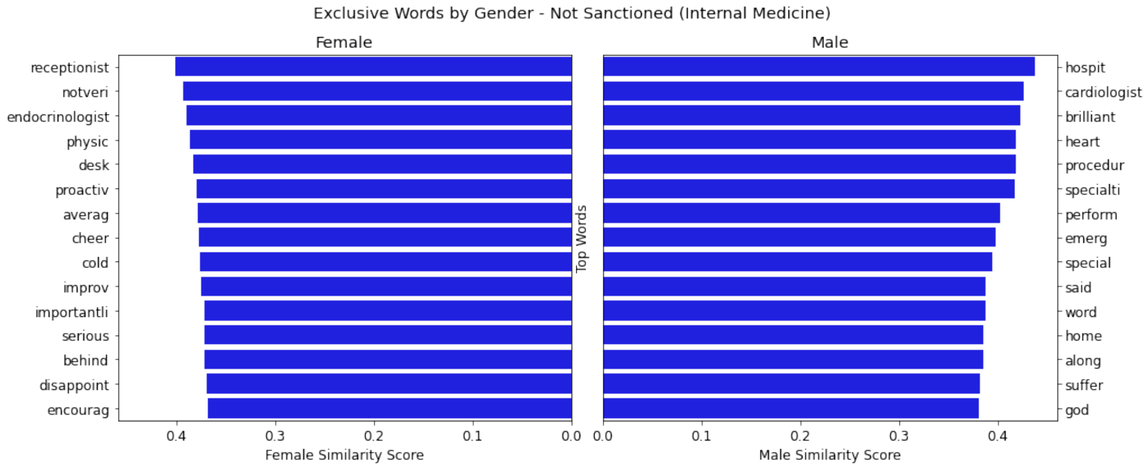

Figure S7: Top words exclusive to unsanctioned men and women for men and women in internal medicine. The x-axis represents the cosine similarity score between all of the words listed on the y categorical axis and the female subset of reviews (left pane) and male subset of reviews (right pane).

S4 OBGYN Results

We analyzed OBGYN reviews for two reasons: (1) they are the third most common type of review after internal medicine and family practice, and (2) we can assume that the majority of the reviewers were women, and we wanted to analyze whether reviewer gender had a significant impact on the reviews. Since the results were largely similar to those of internal medicine, we include them in the Appendix as evidence.

Figures A7 through A11 contain the same types of plots as included in the main text but instead with OBGYN ratings as opposed to Internal Medicine ratings. These plots are the top words by difference in cosine similarity score to the document vector associated with the concatenated reviews of female and male physicians on the left and right panes, respectively, for (A7) the entire OBGYN corpus, (A8) highly ranked reviews, (A9) low-medium ranked reviews, (A10) sanctioned doctors, and (A11) unsanctioned doctors. Figures A12 through A16 contain the same type of plots included above in Appendix S3 but for OBGYN ratings instead of Internal Medicine. These are exclusive words to male and female doctors ranked by similarity score to the document vector of female concatenated reviews and male vector of concatenated reviews respectively for (A12) the entire corpus, (A13) highly ranked reviews, (A14) low-medium ranked reviews, (A15) sanctioned doctors, and (A16) unsanctioned doctors.

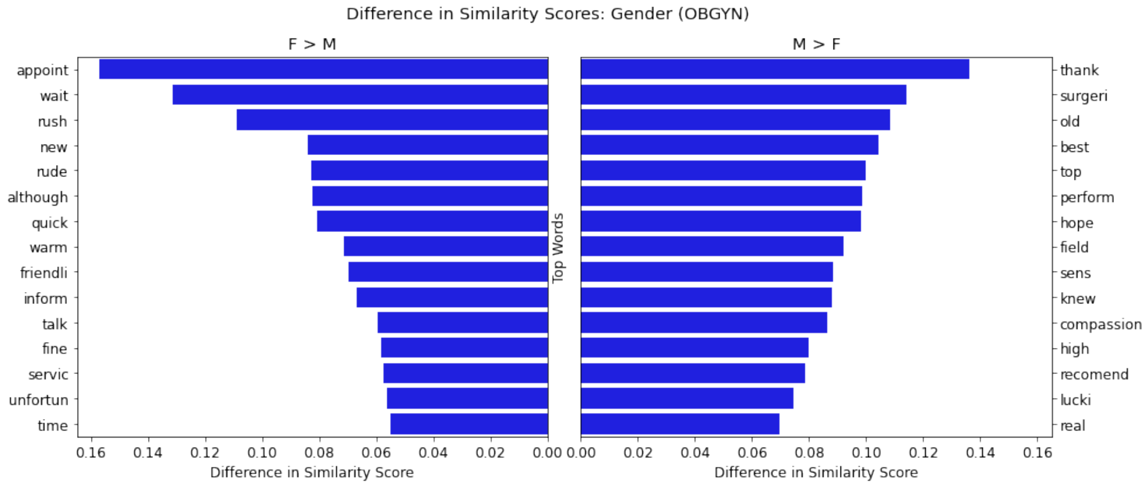

Figure S8: Difference in similarity scores for top words for men and women in OBGYN. TThe x-axis represents the absolute difference in similarity score for the given words to the document vector of concatenated reviews for all women and all men. The figure displays the top 15 words with the biggest differences in similarity scores for the (left pane) female subset of reviews over male reviews and (right pane) male subset of reviews over female subset of reviews.

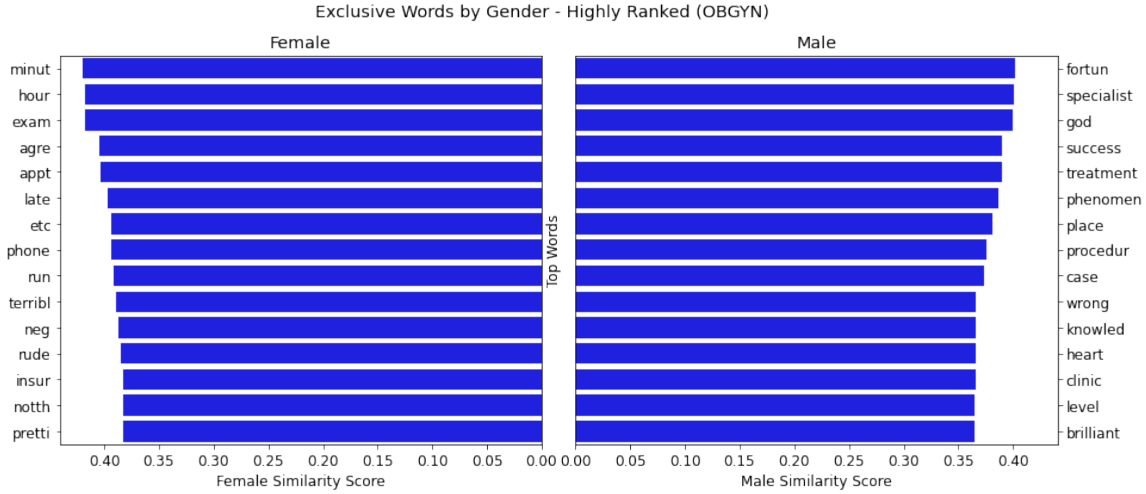

Figure S9: Difference in similarity scores for top words for highly ranked men and women in OBGYN. The x-axis represents the absolute difference in similarity score for the given words to the document vector of concatenated reviews for all highly ranked women and men. The figure displays the top 15 words with the biggest differences in similarity scores for the (left pane) female subset of reviews over male reviews and (right pane) male subset of reviews over female subset of reviews.

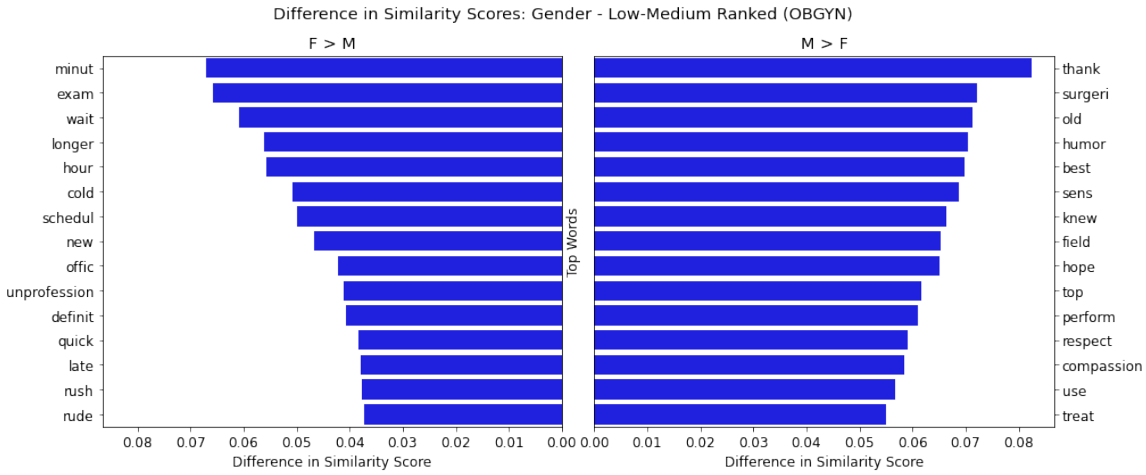

Figure S10: Difference in similarity scores for top words for low-medium ranked men and women in OBGYN. The x-axis represents the absolute difference in similarity score for the given words to the document vector of concatenated reviews for all low-medium ranked women and men. The figure displays the top 15 words with the biggest differences in similarity scores for the (left pane) female subset of reviews over male reviews and (right pane) male subset of reviews over female subset of reviews.

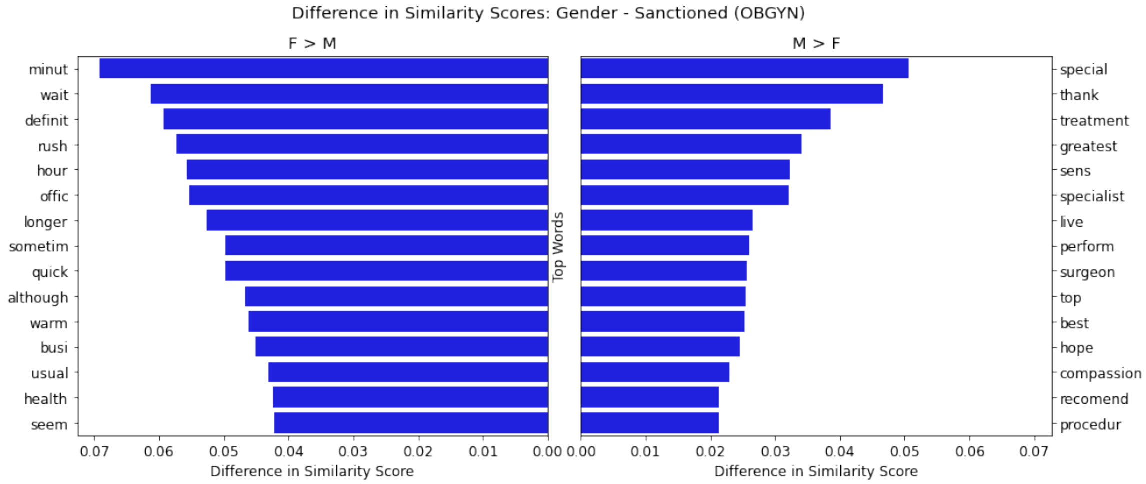

Figure S11: Difference in similarity scores for top words for sanctioned men and women in OBGYN. The x-axis represents the absolute difference in similarity score for the given words to the document vector of concatenated reviews for all sanctioned women and all men. The figure displays the top 15 words with the biggest differences in similarity scores for the (left pane) female subset of reviews over male reviews and (right pane) male subset of reviews over female subset of reviews.

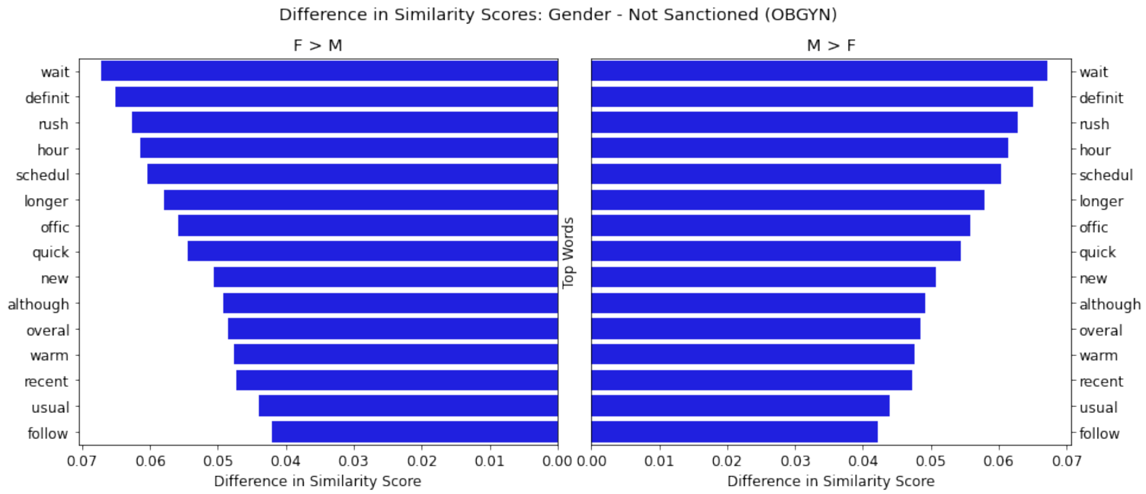

Figure S12: Difference in similarity scores for top words for unsanctioned men and women in OBGYN. The x-axis represents the absolute difference in similarity score for the given words to the document vector of concatenated reviews for all unsanctioned women and all men. The figure displays the top 15 words with the biggest differences in similarity scores for the (left pane) female subset of reviews over male reviews and (right pane) male subset of reviews over female subset of reviews.

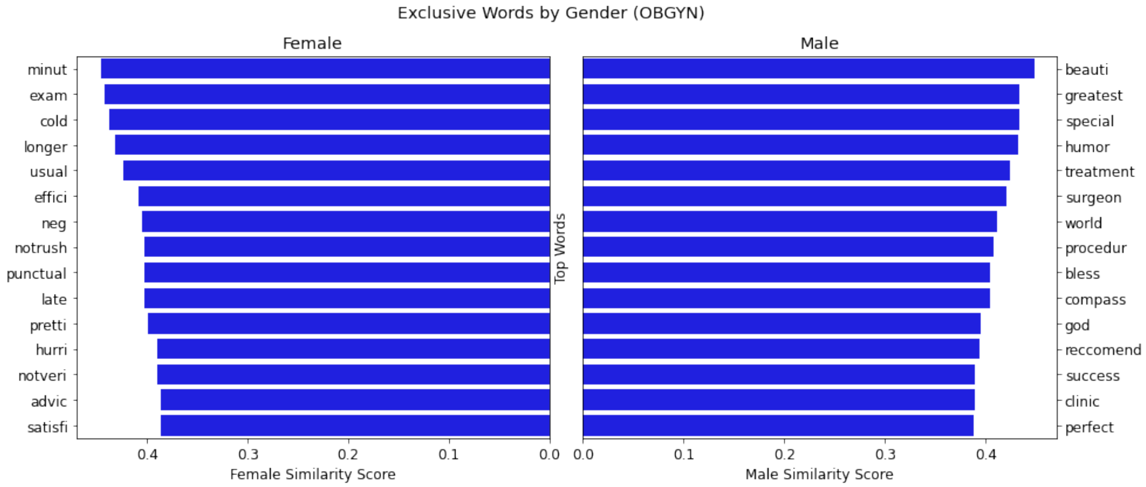

Figure S13: Top words exclusive to men and women for men and women in OBGYN. The x-axis represents the cosine similarity score between all of the words listed on the y categorical axis and the female subset of reviews (left pane) and male subset of reviews (right pane).

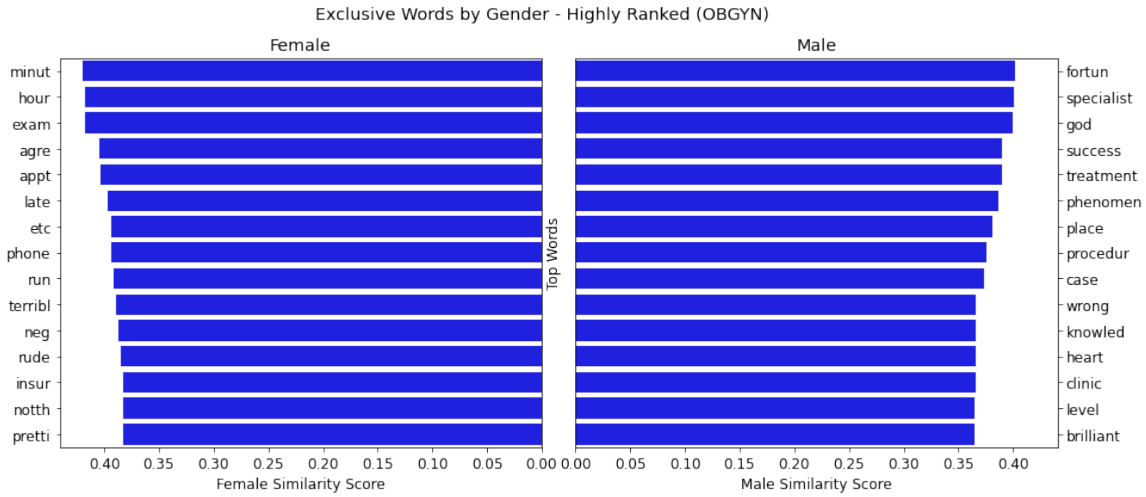

Figure S14: Top words exclusive to highly ranked men and women for men and women in OBGYN. The x-axis represents the cosine similarity score between all of the words listed on the y categorical axis and the female subset of reviews (left pane) and male subset of reviews (right pane).

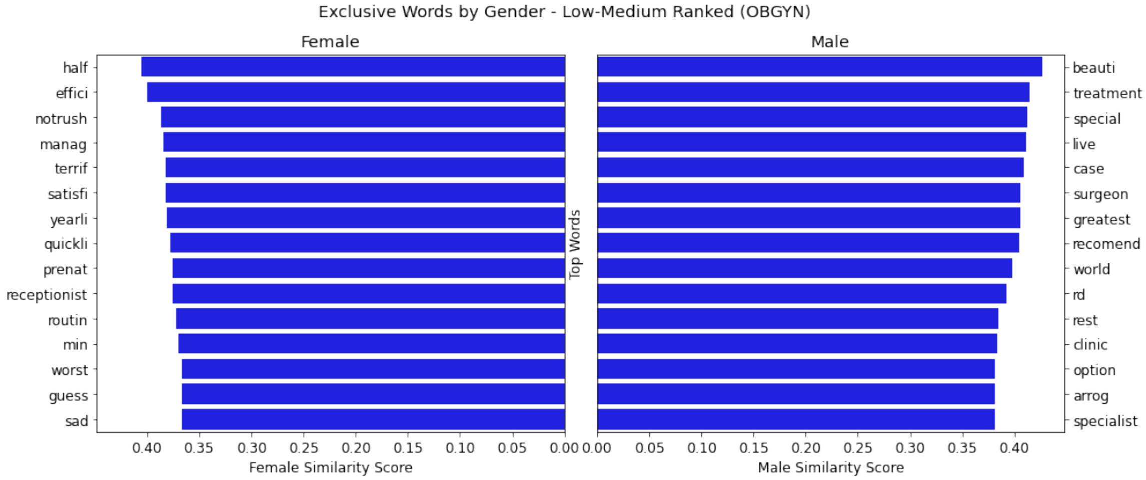

Figure S15: Top words exclusive to low-medium ranked men and women for men and women in OBGYN. The x-axis represents the cosine similarity score between all of the words listed on the y categorical axis and the female subset of reviews (left pane) and male subset of reviews (right pane).

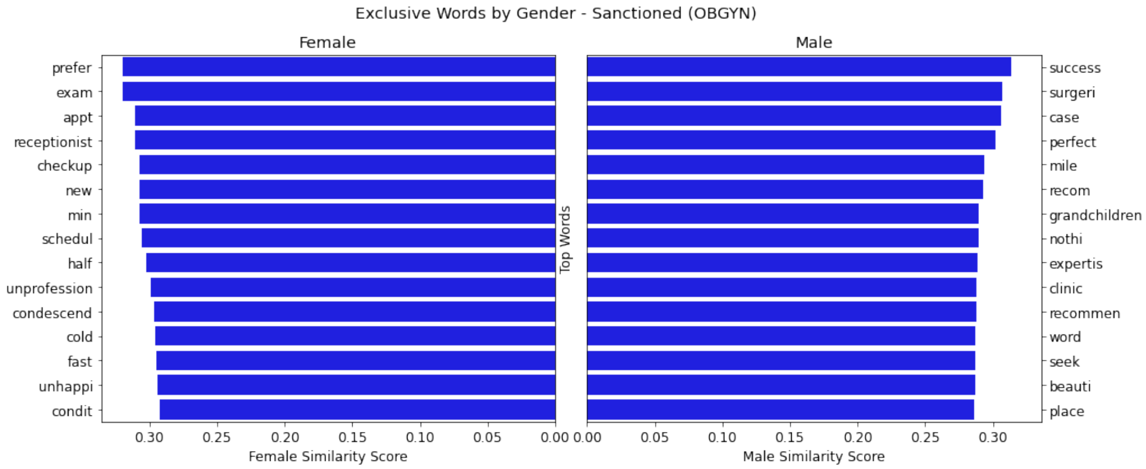

Figure S16: Top words exclusive to sanctioned men and women for men and women in OBGYN. The x-axis represents the cosine similarity score between all of the words listed on the y categorical axis and the female subset of reviews (left pane) and male subset of reviews (right pane).

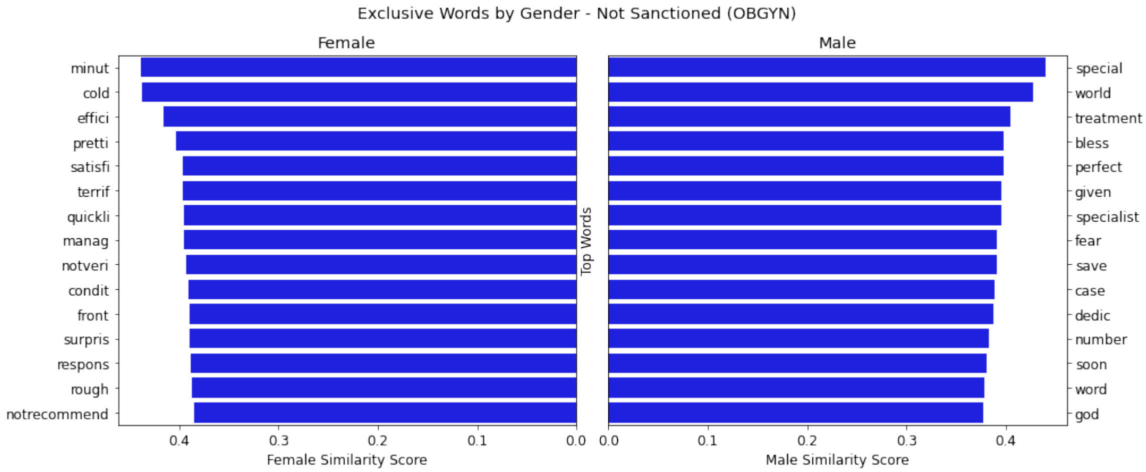

Figure S17: Top words exclusive to unsanctioned men and women for men and women in OBGYN. The x-axis represents the cosine similarity score between all of the words listed on the y categorical axis and the female subset of reviews (left pane) and male subset of reviews (right pane).

S5 Additional Emotion Scoring Output

Figure S18 contains an additional plot from the emotional analysis in which we hold sanction status constant and analyze across genders. This shows us the differences in reviews for (un)sanctioned women and (un)sanctioned men.

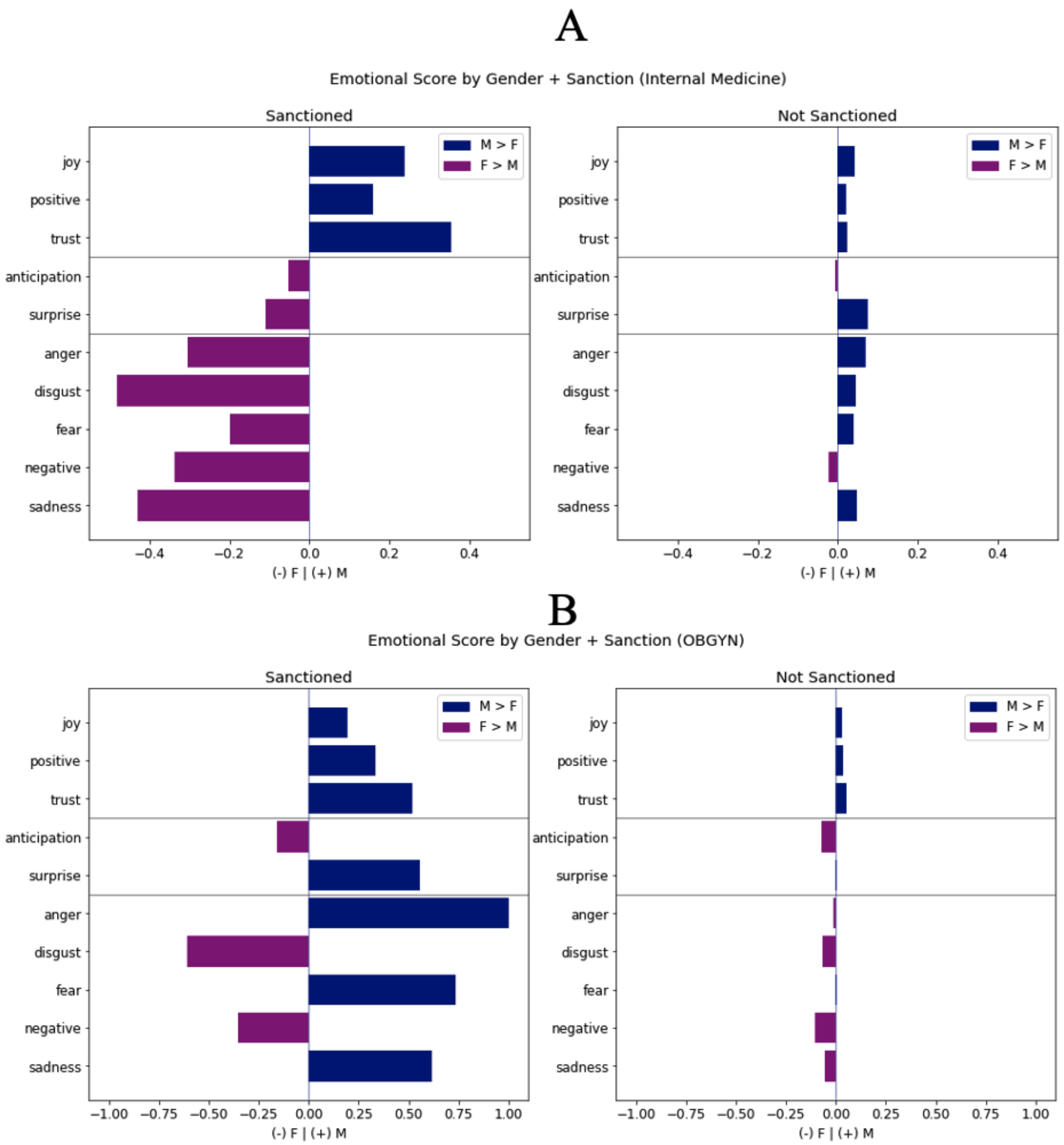

Figure S18: Emotional scores by gender and sanction status for (A) doctors in internal medicine and (B) OBGYNs.

S6 Extended Output from Paragraph Vector Model: Top 300 Words

	Paragraph Vector Model Output Similarity Score						
		Gender		Ranking		Sanction	
	**Word**	**Female**	**Male**	**High**	**Low-Med**	**Sanc.**	**Not Sanc.**
1	**dr**	0.731	0.814	0.795	0.777	0.551	0.855
2	**doctor**	0.706	0.799	0.700	0.767	0.550	0.829
3	**care**	0.632	0.726	0.761	0.647	0.498	0.750
4	**patient**	0.620	0.717	0.618	0.716	0.472	0.740
5	**knowledg**	0.619	0.710	0.730	0.664	0.455	0.738
6	**kind**	0.587	0.692	0.767	0.592	0.450	0.709
7	**time**	0.633	0.684	0.637	0.684	0.463	0.721
8	**great**	0.535	0.681	0.784	0.575	0.418	0.688
9	**one**	0.589	0.681	0.607	0.663	0.454	0.703
10	**person**	0.629	0.679	0.663	0.637	0.474	0.716
11	**help**	0.601	0.679	0.688	0.634	0.461	0.699
12	**extrem**	0.597	0.665	0.575	0.643	0.425	0.711
13	**well**	0.547	0.664	0.671	0.610	0.499	0.680
14	**good**	0.546	0.656	0.661	0.638	0.403	0.672
15	**need**	0.573	0.652	0.635	0.627	0.430	0.676
16	**staff**	0.619	0.648	0.538	0.657	0.487	0.683
17	**wonder**	0.538	0.647	0.735	0.549	0.446	0.660
18	**go**	0.580	0.645	0.582	0.671	0.421	0.673
19	**excel**	0.501	0.643	0.767	0.512	0.388	0.641
20	**realli**	0.597	0.628	0.695	0.574	0.448	0.666
21	**take**	0.587	0.625	0.631	0.604	0.475	0.657
22	**see**	0.619	0.622	0.570	0.656	0.483	0.670
23	**profession**	0.535	0.620	0.660	0.566	0.393	0.652
24	**listen**	0.592	0.620	0.664	0.568	0.400	0.656
25	**know**	0.539	0.620	0.609	0.601	0.412	0.643
26	**offic**	0.612	0.617	0.507	0.673	0.443	0.675
27	**recommend**	0.513	0.616	0.684	0.530	0.373	0.634
28	**physician**	0.534	0.616	0.583	0.575	0.407	0.637
29	**never**	0.582	0.613	0.535	0.653	0.455	0.652
30	**mani**	0.513	0.608	0.576	0.581	0.396	0.603
31	**concern**	0.577	0.606	0.587	0.593	0.405	0.633
32	**manner**	0.470	0.603	0.519	0.556	0.356	0.611
33	**alway**	0.587	0.598	0.689	0.567	0.510	0.646
34	**found**	0.520	0.593	0.525	0.588	0.369	0.620
35	**year**	0.505	0.590	0.577	0.553	0.385	0.601
36	**compassion**	0.512	0.586	0.725	0.472	0.404	0.604
37	**highli**	0.495	0.582	0.730	0.477	0.319	0.593
38	**problem**	0.502	0.579	0.546	0.577	0.382	0.591
39	**best**	0.474	0.575	0.744	0.461	0.369	0.574
40	**anyon**	0.489	0.571	0.524	0.510	0.319	0.580
41	**think**	0.531	0.571	0.517	0.610	0.352	0.602
42	**friendli**	0.551	0.568	0.653	0.529	0.355	0.624
43	**talk**	0.523	0.568	0.524	0.553	0.345	0.601
44	**seen**	0.553	0.559	0.493	0.581	0.404	0.604
45	**thorough**	0.545	0.557	0.713	0.486	0.370	0.608
46	**way**	0.477	0.555	0.517	0.531	0.408	0.568
47	**find**	0.517	0.554	0.551	0.544	0.369	0.582
48	**understand**	0.500	0.553	0.614	0.485	0.335	0.577
49	**medic**	0.477	0.553	0.456	0.581	0.394	0.565
50	**answer**	0.511	0.552	0.558	0.537	0.372	0.580

51	**intellig**	0.475	0.550	0.582	0.480	0.326	0.556
52	**even**	0.507	0.550	0.458	0.601	0.318	0.578
53	**say**	0.491	0.549	0.485	0.560	0.393	0.578
54	**thought**	0.505	0.548	0.489	0.534	0.279	0.573
55	**work**	0.499	0.544	0.505	0.542	0.330	0.592
56	**feel**	0.521	0.536	0.560	0.513	0.354	0.568
57	**question**	0.494	0.535	0.494	0.520	0.331	0.564
58	**give**	0.489	0.531	0.479	0.535	0.344	0.551
59	**make**	0.513	0.531	0.521	0.526	0.379	0.576
60	**love**	0.476	0.529	0.638	0.464	0.431	0.551
61	**seem**	0.528	0.527	0.420	0.607	0.336	0.560
62	**much**	0.495	0.526	0.527	0.537	0.377	0.563
63	**follow**	0.489	0.524	0.518	0.495	0.331	0.554
64	**actual**	0.523	0.521	0.513	0.540	0.359	0.564
65	**ask**	0.470	0.512	0.402	0.555	0.320	0.539
66	**nurs**	0.470	0.511	0.446	0.525	0.408	0.537
67	**first**	0.482	0.511	0.476	0.526	0.353	0.531
68	**thing**	0.483	0.509	0.514	0.508	0.346	0.539
69	**lot**	0.482	0.501	0.534	0.493	0.343	0.531
70	**polit**	0.471	0.493	0.490	0.480	0.388	0.506
71	**experi**	0.471	0.492	0.400	0.532	0.329	0.522
72	**rush**	0.499	0.490	0.490	0.502	0.356	0.543
73	**health**	0.482	0.480	0.464	0.502	0.393	0.514
74	**spend**	0.491	0.473	0.492	0.484	0.315	0.518
75	**issu**	0.488	0.472	0.473	0.491	0.331	0.508
76	**attent**	0.476	0.468	0.517	0.448	0.393	0.504
77	**last**	0.484	0.465	0.408	0.520	0.326	0.503
78	**punctual**	0.476	0.465	0.506	0.456	0.337	0.488
79	**wait**	0.486	0.460	0.404	0.534	0.389	0.516
80	**primari**	0.474	0.459	0.474	0.426	0.318	0.492
81	**chang**	0.474	0.447	0.425	0.481	0.356	0.491
82	**appoint**	0.490	0.446	0.396	0.506	0.319	0.497
83	**pleasant**	0.468	0.527	0.563	0.497	0.311	0.550
84	**long**	0.468	0.463	0.432	0.516	0.385	0.502
85	**visit**	0.467	0.478	0.434	0.501	0.295	0.506
86	**compet**	0.466	0.538	0.522	0.519	0.316	0.551
87	**look**	0.465	0.477	0.430	0.525	0.330	0.523
88	**want**	0.463	0.550	0.470	0.603	0.373	0.580
89	**warm**	0.462	0.451	0.553	0.414	0.307	0.501
90	**happi**	0.461	0.476	0.587	0.442	0.321	0.517
91	**new**	0.460	0.466	0.459	0.485	0.291	0.510
92	**everi**	0.460	0.505	0.486	0.494	0.323	0.538
93	**call**	0.458	0.499	0.467	0.503	0.329	0.541
94	**come**	0.458	0.510	0.446	0.533	0.330	0.544
95	**may**	0.457	0.496	0.439	0.541	0.342	0.507
96	**nice**	0.456	0.555	0.537	0.547	0.309	0.575
97	**notonli**	0.456	0.577	0.507	0.485	0.370	0.564
98	**truli**	0.455	0.530	0.648	0.426	0.357	0.547
99	**far**	0.455	0.497	0.469	0.500	0.358	0.516
100	**everyon**	0.455	0.503	0.522	0.493	0.349	0.542
101	**back**	0.454	0.491	0.421	0.542	0.320	0.525
102	**refer**	0.454	0.500	0.498	0.474	0.327	0.523
103	**took**	0.447	0.539	0.526	0.505	0.378	0.557
104	**bedsid**	0.447	0.566	0.482	0.522	0.322	0.562
105	**past**	0.446	0.469	0.429	0.511	0.319	0.506
106	**deal**	0.444	0.463	0.375	0.502	0.304	0.509
107	**practic**	0.443	0.470	0.419	0.503	0.375	0.494
108	**explain**	0.443	0.520	0.544	0.448	0.299	0.536
109	**respons**	0.443	0.441	0.404	0.475	0.327	0.489

110	**usual**	0.443	0.404	0.426	0.442	0.359	0.446
111	**anyth**	0.442	0.498	0.417	0.529	0.273	0.521
112	**sometim**	0.442	0.422	0.413	0.457	0.353	0.458
113	**everyth**	0.442	0.541	0.574	0.463	0.289	0.554
114	**assist**	0.441	0.410	0.377	0.451	0.330	0.462
115	**get**	0.441	0.516	0.478	0.525	0.298	0.545
116	**almost**	0.441	0.492	0.400	0.534	0.369	0.503
117	**still**	0.441	0.528	0.457	0.524	0.328	0.529
118	**definit**	0.441	0.480	0.481	0.462	0.286	0.509
119	**smart**	0.440	0.507	0.550	0.452	0.356	0.523
120	**better**	0.440	0.529	0.534	0.499	0.313	0.535
121	**sever**	0.440	0.566	0.460	0.573	0.347	0.560
122	**import**	0.438	0.446	0.423	0.455	0.325	0.482
123	**detail**	0.438	0.474	0.540	0.414	0.195	0.516
124	**wish**	0.438	0.466	0.493	0.422	0.353	0.499
125	**tell**	0.438	0.468	0.403	0.524	0.309	0.496
126	**anoth**	0.438	0.512	0.418	0.550	0.298	0.527
127	**tri**	0.438	0.474	0.408	0.535	0.300	0.498
128	**except**	0.436	0.517	0.477	0.478	0.390	0.508
129	**leav**	0.433	0.407	0.386	0.431	0.305	0.445
130	**respect**	0.433	0.514	0.562	0.462	0.319	0.537
131	**phone**	0.432	0.425	0.373	0.465	0.351	0.454
132	**genuin**	0.431	0.459	0.579	0.388	0.307	0.482
133	**treat**	0.431	0.558	0.445	0.558	0.430	0.563
134	**gone**	0.430	0.428	0.444	0.427	0.287	0.450
135	**inform**	0.430	0.494	0.420	0.501	0.325	0.518
136	**right**	0.429	0.488	0.459	0.477	0.334	0.508
137	**run**	0.429	0.440	0.345	0.490	0.336	0.487
138	**littl**	0.427	0.469	0.420	0.516	0.296	0.486
139	**previou**	0.426	0.389	0.359	0.420	0.266	0.423
140	**effici**	0.426	0.456	0.556	0.401	0.321	0.481
141	**internist**	0.426	0.429	0.497	0.395	0.199	0.463
142	**day**	0.424	0.498	0.470	0.495	0.338	0.508
143	**notrush**	0.424	0.426	0.532	0.384	0.373	0.471
144	**especi**	0.424	0.430	0.358	0.458	0.264	0.461
145	**consider**	0.423	0.494	0.510	0.441	0.346	0.510
146	**easi**	0.423	0.464	0.583	0.372	0.298	0.476
147	**discuss**	0.422	0.391	0.380	0.434	0.490	0.447
148	**receptionist**	0.422	0.134	0.156	0.396	0.276	0.384
149	**diagnosi**	0.422	0.486	0.415	0.452	0.146	0.482
150	**nota**	0.422	0.475	0.398	0.514	0.366	0.505
151	**interest**	0.420	0.466	0.423	0.471	0.278	0.489
152	**someth**	0.420	0.401	0.404	0.448	0.260	0.445
153	**result**	0.419	0.428	0.379	0.476	0.278	0.480
154	**provid**	0.419	0.457	0.419	0.454	0.347	0.480
155	**switch**	0.418	0.374	0.362	0.417	0.272	0.407
156	**incred**	0.418	0.438	0.426	0.409	0.159	0.471
157	**went**	0.417	0.496	0.415	0.537	0.286	0.524
158	**without**	0.416	0.488	0.399	0.498	0.347	0.478
159	**hard**	0.415	0.409	0.412	0.427	0.270	0.444
160	**peopl**	0.415	0.474	0.357	0.519	0.360	0.489
161	**pleas**	0.415	0.472	0.403	0.483	0.284	0.492
162	**treatment**	0.415	0.512	0.460	0.498	0.378	0.514
163	**saw**	0.415	0.478	0.401	0.517	0.253	0.491
164	**rude**	0.414	0.422	0.149	0.584	0.277	0.462
165	**busi**	0.414	0.489	0.429	0.512	0.379	0.509
166	**differ**	0.413	0.470	0.432	0.487	0.286	0.483
167	**often**	0.413	0.377	0.334	0.445	0.317	0.414
168	**keep**	0.413	0.470	0.464	0.452	0.343	0.487

169	**specialist**	0.413	0.436	0.453	0.416	0.312	0.462
170	**gener**	0.413	0.447	0.394	0.475	0.334	0.454
171	**address**	0.412	0.398	0.366	0.430	0.335	0.425
172	**complet**	0.410	0.473	0.330	0.494	0.296	0.492
173	**reason**	0.410	0.452	0.366	0.518	0.396	0.492
174	**other**	0.409	0.456	0.425	0.450	0.314	0.460
175	**minut**	0.409	0.419	0.346	0.484	0.288	0.451
176	**famili**	0.408	0.546	0.559	0.471	0.402	0.539
177	**impress**	0.408	0.408	0.473	0.410	0.194	0.434
178	**return**	0.406	0.476	0.398	0.485	0.349	0.485
179	**test**	0.405	0.445	0.380	0.482	0.318	0.478
180	**overal**	0.405	0.419	0.417	0.416	0.341	0.439
181	**got**	0.404	0.470	0.432	0.510	0.338	0.492
182	**current**	0.404	0.424	0.409	0.443	0.246	0.469
183	**pcp**	0.404	0.362	0.397	0.385	0.192	0.411
184	**experienc**	0.404	0.430	0.353	0.463	0.261	0.456
185	**although**	0.403	0.462	0.389	0.481	0.287	0.481
186	**felt**	0.403	0.421	0.372	0.445	0.273	0.447
187	**approach**	0.403	0.438	0.460	0.394	0.197	0.446
188	**will**	0.403	0.431	0.549	0.400	0.371	0.450
189	**diagnos**	0.403	0.481	0.422	0.466	0.309	0.474
190	**hurri**	0.402	0.401	0.338	0.442	0.197	0.439
191	**courteou**	0.401	0.480	0.550	0.428	0.313	0.490
192	**appropri**	0.401	0.353	0.427	0.315	0.297	0.384
193	**spent**	0.400	0.415	0.394	0.424	0.192	0.447
194	**fantast**	0.399	0.435	0.607	0.359	0.319	0.462
195	**review**	0.399	0.365	0.311	0.420	0.278	0.402
196	**left**	0.399	0.381	0.295	0.474	0.317	0.415
197	**though**	0.399	0.447	0.357	0.488	0.263	0.469
198	**less**	0.399	0.394	0.282	0.463	0.270	0.427
199	**someon**	0.399	0.416	0.393	0.473	0.324	0.453
200	**amp**	0.399	0.425	0.372	0.431	0.305	0.450
201	**knowleg**	0.399	0.476	0.492	0.424	0.340	0.485
202	**individu**	0.399	0.422	0.406	0.418	0.268	0.442
203	**bad**	0.399	0.421	0.341	0.491	0.303	0.438
204	**doc**	0.398	0.487	0.490	0.469	0.319	0.508
205	**use**	0.397	0.533	0.437	0.523	0.334	0.520
206	**abl**	0.397	0.465	0.528	0.417	0.264	0.467
207	**attitud**	0.397	0.402	0.390	0.476	0.296	0.449
208	**total**	0.396	0.446	0.395	0.497	0.333	0.459
209	**made**	0.395	0.448	0.369	0.466	0.292	0.475
210	**matter**	0.394	0.428	0.424	0.422	0.268	0.440
211	**fact**	0.393	0.396	0.327	0.470	0.279	0.417
212	**turn**	0.393	0.388	0.373	0.443	0.323	0.403
213	**around**	0.393	0.469	0.444	0.434	0.372	0.473
214	**trust**	0.392	0.470	0.520	0.418	0.279	0.491
215	**entir**	0.392	0.461	0.365	0.441	0.298	0.459
216	**like**	0.392	0.457	0.406	0.430	0.287	0.459
217	**field**	0.392	0.483	0.476	0.441	0.287	0.489
218	**woman**	0.391	0.116	0.286	0.398	0.219	0.377
219	**front**	0.391	0.379	0.285	0.427	0.297	0.415
220	**condit**	0.391	0.490	0.424	0.477	0.294	0.487
221	**support**	0.391	0.400	0.423	0.379	0.302	0.416
222	**goe**	0.391	0.428	0.470	0.378	0.254	0.442
223	**final**	0.390	0.382	0.290	0.445	0.283	0.424
224	**addit**	0.390	0.417	0.357	0.436	0.259	0.446
225	**hour**	0.390	0.413	0.339	0.464	0.286	0.444
226	**two**	0.390	0.482	0.376	0.522	0.290	0.493
227	**check**	0.390	0.413	0.407	0.426	0.287	0.434

228	**schedul**	0.389	0.406	0.359	0.440	0.193	0.436
229	**empathet**	0.389	0.399	0.428	0.371	0.194	0.434
230	**referr**	0.389	0.366	0.415	0.394	0.313	0.401
231	**ill**	0.389	0.417	0.374	0.425	0.326	0.417
232	**hand**	0.388	0.448	0.427	0.435	0.295	0.440
233	**gave**	0.387	0.453	0.397	0.467	0.265	0.475
234	**md**	0.387	0.407	0.368	0.420	0.309	0.442
235	**amaz**	0.387	0.457	0.573	0.377	0.342	0.460
236	**rare**	0.387	0.407	0.455	0.379	0.309	0.409
237	**examin**	0.387	0.422	0.328	0.452	0.295	0.433
238	**difficult**	0.385	0.396	0.368	0.425	0.292	0.420
239	**continu**	0.385	0.472	0.421	0.471	0.281	0.481
240	**possibl**	0.384	0.460	0.423	0.431	0.291	0.478
241	**bit**	0.384	0.404	0.373	0.434	0.193	0.432
242	**yet**	0.383	0.435	0.381	0.448	0.255	0.454
243	**comfort**	0.383	0.476	0.589	0.372	0.305	0.471
244	**insur**	0.383	0.403	0.364	0.435	0.263	0.434
245	**glad**	0.382	0.416	0.459	0.381	0.323	0.423
246	**met**	0.382	0.524	0.485	0.437	0.305	0.517
247	**enough**	0.381	0.485	0.476	0.445	0.293	0.488
248	**worth**	0.380	0.396	0.458	0.373	0.315	0.417
249	**came**	0.380	0.438	0.379	0.451	0.287	0.460
250	**friend**	0.380	0.482	0.508	0.404	0.330	0.480
251	**longer**	0.379	0.392	0.381	0.423	0.322	0.410
252	**complaint**	0.379	0.418	0.329	0.474	0.310	0.439
253	**encount**	0.379	0.411	0.299	0.394	0.274	0.420
254	**top**	0.378	0.461	0.474	0.413	0.270	0.474
255	**exam**	0.378	0.375	0.478	0.434	0.268	0.399
256	**diagnostician**	0.378	0.487	0.521	0.410	0.296	0.490
257	**sure**	0.377	0.442	0.518	0.375	0.297	0.445
258	**awesom**	0.376	0.458	0.586	0.375	0.334	0.478
259	**recent**	0.376	0.440	0.448	0.440	0.281	0.462
260	**nothav**	0.375	0.446	0.395	0.476	0.396	0.453
261	**whole**	0.375	0.419	0.423	0.377	0.357	0.432
262	**show**	0.374	0.427	0.342	0.475	0.285	0.446
263	**short**	0.374	0.394	0.326	0.426	0.289	0.401
264	**prompt**	0.374	0.414	0.496	0.382	0.253	0.439
265	**necessari**	0.373	0.427	0.462	0.378	0.279	0.431
266	**area**	0.372	0.455	0.448	0.407	0.314	0.455
267	**avail**	0.371	0.452	0.474	0.404	0.312	0.464
268	**hear**	0.371	0.354	0.362	0.402	0.292	0.401
269	**might**	0.371	0.386	0.385	0.424	0.386	0.394
270	**promptli**	0.371	0.394	0.435	0.364	0.283	0.420
271	**believ**	0.370	0.473	0.374	0.497	0.320	0.468
272	**quick**	0.370	0.408	0.380	0.429	0.262	0.424
273	**probabl**	0.369	0.464	0.378	0.479	0.273	0.452
274	**job**	0.369	0.451	0.408	0.458	0.292	0.487
275	**either**	0.366	0.388	0.377	0.479	0.298	0.401
276	**instead**	0.366	0.387	0.377	0.465	0.276	0.403
277	**commun**	0.365	0.449	0.400	0.433	0.279	0.466
278	**horribl**	0.365	0.378	0.286	0.478	0.387	0.393
279	**etc**	0.365	0.369	0.338	0.420	0.327	0.413
280	**taken**	0.364	0.470	0.426	0.470	0.385	0.477
281	**plan**	0.363	0.414	0.410	0.383	0.257	0.422
282	**manag**	0.363	0.405	0.459	0.429	0.339	0.424
283	**skill**	0.363	0.491	0.402	0.455	0.287	0.480
284	**stuff**	0.362	0.410	0.409	0.383	0.306	0.416
285	**number**	0.362	0.349	0.339	0.383	0.194	0.392
286	**full**	0.361	0.396	0.352	0.415	0.269	0.421

287	**month**	0.361	0.368	0.365	0.440	0.256	0.401
288	**done**	0.361	0.455	0.408	0.454	0.257	0.464
289	**twice**	0.359	0.374	0.358	0.429	0.190	0.401
290	**clearli**	0.358	0.443	0.350	0.430	0.258	0.448
291	**plu**	0.357	0.405	0.375	0.389	0.298	0.417
292	**cold**	0.357	0.321	0.368	0.455	0.395	0.361
293	**mind**	0.357	0.397	0.360	0.414	0.288	0.421
294	**dedic**	0.354	0.451	0.586	0.379	0.331	0.450
295	**earth**	0.352	0.510	0.575	0.381	0.310	0.499
296	**next**	0.352	0.395	0.372	0.403	0.319	0.412
297	**order**	0.351	0.389	0.442	0.422	0.300	0.411
298	**medicin**	0.351	0.466	0.398	0.453	0.315	0.459
299	**late**	0.351	0.381	0.325	0.441	0.192	0.413
300	**sick**	0.349	0.390	0.340	0.395	0.338	0.394

Figure S19: Top 300 words (ranked by female score) for internal medicine paragraph vector model outputs. We find the best hyper-parameter setting producing interpretable results is vector size of 300, window size of 30, minimum counts of 30, and negative sampling drawing 7 noise words. Based on this setting, we set the hyper-parameter of doc2vec to be (dm = 0, window = 30, vector size = 300, min count = 30, epochs = 100, workers = 4, hs = 0, negative = 7, dbow words = 1, dm concat = 1).
